# High‐Throughput Strategies for Streamlining Lipid Nanoparticle Development Pipeline

**DOI:** 10.1002/advs.202511551

**Published:** 2025-10-03

**Authors:** Lois Lam, Stephanie Watson, Yogambha Ramaswamy, Gurvinder Singh

**Affiliations:** ^1^ The School of Biomedical Engineering Faculty of IT and Engineering Sydney Nano Institute The University of Sydney Camperdown New South Wales 2008 Australia; ^2^ Sydney Medical School Faculty of Medicine and Health Sydney Nano Institute The University of Sydney Camperdown New South Wales 2008 Australia

**Keywords:** barcoding strategies, closed‐loop workflow, high‐throughput screening, lipid nanoparticles, machine learning, therapeutic delivery

## Abstract

Lipid nanoparticles (LNPs) have become clinically validated nanocarriers for nucleic acid delivery, enabling applications in mRNA vaccines and therapies for cancer, ocular, and infectious diseases. Identifying LNPs formulations with optimal physicochemical and pharmacokinetic properties using traditional low‐throughput methods is resource‐intensive and impractical for evaluating large libraries. Recent advances in automation, high‐throughput platforms for lipid synthesis, characterization, and screening tools are transforming the landscape of LNP formulation. These strategies enable rapid multi‐parametric generation and evaluation of hundreds to thousands of formulations across key properties such as size, charge, stability, biodistribution, cellular uptake, and intracellular trafficking. In parallel, advanced biomimetic models and in vivo multiplexed barcoding screening strategies provide deeper insights into tissue targeting and therapeutic delivery outcomes. This review provides an integrated framework that combines automation with high‐throughput combinatorial synthesis, characterization, and in vitro/in vivo screening tools. In this development pipeline, performance benchmarks applied at each step systematically exclude suboptimal candidates, ensuring that only clinically viable LNP candidates advance. Future directions, including automation, high‐throughput, and closed‐loop machine learning guided design strategies, are further discussed to advance the development of next‐generation LNP therapeutics and accelerate their translation from bench to bedside.

## Graphical abstract

This review highlights emerging state‐of‐the‐art high‐throughput strategies for optimising lipid nanoparticle formulation. By integrating combinatorial design, characterization, in vitro/in vivo screening, automation, and machine learning into a closed‐loop framework, it provides a roadmap to streamline discovery and accelerate the translation of clinically viable lipid nanoparticles for applications from vaccines to therapies.

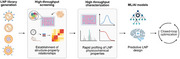

## 1 Introduction

Nanoparticles (NPs) have redefined the landscape of therapeutic delivery by enabling precise targeting of biological sites, thereby improving both therapeutic efficacy and treatment outcomes. While traditional drug formulations have relied on small molecules, recent advances in nanotechnology have expanded the scope of therapeutics to include proteins, peptides, nucleic acids, and monoclonal antibodies. This expanded scope of therapeutics has unlocked new therapeutic functionalities, but has also led to new challenges.^[^ ^1^ ^]^ Nucleic acids, for instance, can target the genetic origins of diseases to achieve long‐lasting or even curative effects. Their therapeutic potential is hindered by their rapid degradation and low cellular uptake due to electrostatic repulsion from negatively charged cell membranes.^[^ ^2^ ^]^ NPs, particularly lipid nanoparticles (LNPs), have emerged as a promising carrier to address these challenges, providing enhanced solubility, stability, biodistribution, and pharmacokinetics profiles compared to free‐form therapeutics.^[^ ^3^, ^4^ ^]^ Recent advancements in high‐resolution imaging and analytical techniques have further deepened mechanistic insights not only into the biological and pathological states of diseases^[^ ^5^, ^6^, ^7^, ^8^ ^]^ but also into how their local environment interacts with NPs.^[^ ^9^, ^10^, ^11^ ^]^ Techniques such as high‐resolution microscopy allow direct visualization of NP‐cell interaction,^[^ ^12^ ^]^ while advanced analytical tools help in quantifying NP distribution, stability, and therapeutic release dynamics.^[^ ^1^, ^13^, ^14^ ^]^ These technological developments are driving a paradigm shift from empirical NP formulation design toward precision‐engineered nanocarriers tailored to address therapeutic needs.^[^ ^15^ ^]^

Among various NPs, LNPs are the most common class of Food and Drug Administration (FDA) approved nanocarriers, owing to their excellent biocompatibility, ease in formulation, and tunable physicochemical properties.^[^ ^16^ ^]^ Since the discovery of liposomes in the 1960s,^[^ ^17^ ^]^ LNPs have evolved into more advanced formulations, including the incorporation of polyethylene glycol (PEG) coatings to enhance circulation time by reducing immune recognition,^[^ ^17^, ^18^ ^]^ and the development of pH‐responsive ionizable lipids that facilitate intracellular delivery through endosomal escape.^[^ ^19^, ^20^ ^]^ These advanced LNPs typically integrate four essential components: i) an ionizable lipid to facilitate endosome escape; ii) a PEG‐lipid conjugate to enhance circulation time and stability; iii) cholesterol to improve membrane fusion and structural integrity; and iv) a helper lipid to enhance encapsulation efficiency and therapeutic delivery.^[^ ^19^, ^21^, ^22^ ^]^

**Figure 1:**
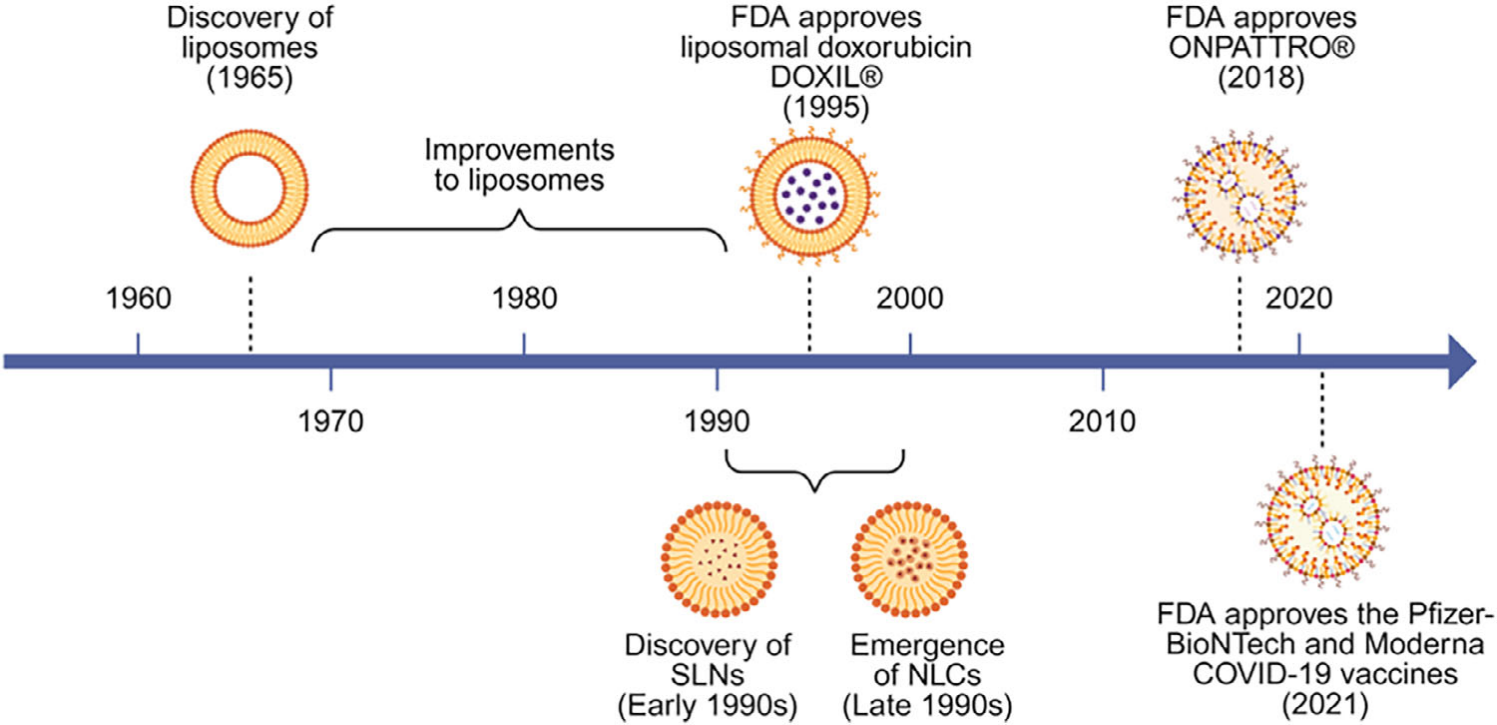
Timeline of LNP‐based therapeutic development. Key milestone in the evolution of lipid‐based nanocarriers, from the early discovery of liposomes to the modern ionizable LNPs that have enabled the clinical translation of mRNA and siRNA therapeutics. Created with BioRender.

Solid lipid nanoparticles (SLNs) developed in the early 1990s (**Figure** **1**), emerged as a distinct class of nanocarriers offering several advantages over liposomes, such as improved long‐term physical stability,^[^ ^23^ ^]^ enhanced drug bioavailability,^[^ ^17^ ^]^ controlled therapeutic release due to the reduced mobility of therapeutic molecules in the solid state,^[^ ^24^ ^]^ and ease in the scalability.^[^ ^25^ ^]^ However, lipid crystallization in SLNs led to drug expulsion during storage,^[^ ^26^ ^]^ a limitation addressed by NLCs (nanostructured lipid carriers), which incorporate a blend of solid and liquid lipids to enhance therapeutic retention and stability. These advances have underpinned the use of SLNs and NLCs in cosmetic and dermal pharmaceutical products,^[^ ^26^, ^27^ ^]^ demonstrating their versatility beyond therapeutics. These benefits have facilitated the successful clinical translation of lipid‐based therapeutics, including the FDA‐approved product Onpattro (Patisiran, 2018), an ionizable LNP for treating hereditary transthyretin‐mediated amyloidosis (hATTR amyloidosis).^[^ ^28^, ^29^ ^]^ More recently, ionizable LNPs have played a crucial role in the rapid development of the Pfizer‐BioNTech and Moderna mRNA COVID‐19 vaccines by effectively delivering mRNA into cells while protecting the mRNA from degradation.^[^ ^17^, ^19^, ^22^ ^]^

Although LNP technology holds great therapeutic potential, the number of clinically approved LNP‐based products remains low relative to the extensive volume of research publications using LNPs. According to a recent report from the European Medicines Agency (EMA), only 14 LNP‐based formulations have been approved by the FDA or EMA, with over 10 additional formulations currently progressing through clinical trials.^[^ ^30^ ^]^ This prolonged timeline in the clinical development of LNPs is partly due to challenges in scalability and batch‐to‐batch reproducibility, both of which are critical for ensuring consistent size, surface charge, and therapeutic loading.^[^ ^31^ ^]^ In recent years, combinatorial lipid chemistry has enabled the synthesis of tens to hundreds of LNP formulations in parallel within short timeframes, facilitating novel lipid discovery for various disease applications. Microfluidic technologies, which allow for the manipulation of fluids at the microscale, offer a cost‐effective solution for efficient LNP synthesis from these lipid libraries, producing monodisperse formulations with superior batch consistency compared to conventional methods.^[^ ^32^, ^33^, ^34^, ^35^ ^]^ Importantly, this method allows researchers to formulate LNP libraries in multi‐well plates (i.e., high‐throughput capability with up to 384 LNPs per plate and many plates per run) with minimal reagent consumption,^[^ ^36^ ^]^ thus facilitating the rapid selection of lead candidates for subsequent evaluation. Nevertheless, addressing synthesis‐related challenges alone is insufficient for accelerating the clinical translation of LNPs. A key bottleneck in advancing LNP development lies in the need for high‐throughput characterization (HTC) and high‐throughput screening (HTS) strategies capable of rapidly identifying a small subset of lead candidates from extensive LNP formulation libraries. Traditional methods for LNP evaluations are inherently low‐throughput due to their reliance on sequential, manual processes that require substantial time, reagents, and operator intervention. For example, techniques such as conventional dynamic light scattering (DLS) and UV–vis spectroscopy necessitate individual sample preparation and measurements, limiting throughput to tens of formulations per day. Such workflows are incompatible with the demands of LNP development pipelines, which often require the screening of hundreds to thousands of candidates generated through combinatorial lipid libraries to identify formulations with optimal physicochemical properties. HTC strategies can overcome these limitations by incorporating automated and parallelized systems that dramatically increase data throughput while significantly reducing sample and reagent consumption. HTC tools, including multi‐well plate (ranging from 96 to 1536 wells) based DLS, spectroscopy, and small‐angle scattering (SAXS) techniques, enable rapid physicochemical profiling (e.g., size, stability, surface charge, encapsulation, and release kinetics of payloads) across hundreds to thousands of LNP formulations. These HTC methods do not provide insights into the biological outcomes and therapeutic efficacies of LNPs. HTS approaches spanning multiplexed in vitro assays and barcoded in vivo studies are increasingly essential to identify LNP candidates with favorable pharmacokinetics, cellular uptake, and tissue‐specific delivery profiles.

Unlike previous reviews that have focused primarily on LNP formulation‐based therapeutic delivery platforms, microfluidic synthesis, and combinatorial design strategies,^[^ ^17^, ^19^, ^32^, ^33^, ^34^, ^37^, ^38^, ^39^, ^40^ ^]^ this review provides a broader perspective on an integrated LNP discovery framework that combines automated synthesis with HTC techniques (e.g., DLS, SAXS, and microplate readers) and HTS strategies. The pipeline begins with combinatorial lipid chemistry, progresses through automated formulation of large LNP libraries, and proceeds to in vitro and in vivo HTS before final physiochemical characterization using HTC and candidate selection. At each stage, sequential decision gates such as thresholds for LNP formulation quality (e.g., reproducibility and yield), acceptable size distribution (20–200 nm), polydispersity index (<0.5), cytotoxicity cutoffs, transfection efficiency, biodistribution, tissue targeting, therapeutic efficacy, encapsulation efficiency, and payload release kinetics, systematically eliminate suboptimal formulations. Incorporating such performance benchmarks within the LNP development pipeline ensures that only candidates with favorable physicochemical, pharmacokinetic, and biological characteristics progress toward preclinical evaluation. This integrated framework allows rapid iteration between formulation and function, reduces experimental burden, and accelerates the identification of clinically viable LNPs. Finally, we provide a forward vision on how high‐throughput and data‐driven strategies can transform LNP development into a predictive, scalable, and clinically translatable framework.

## 2 High‐Throughput Combinatorial Synthesis

Ionizable lipids are central to the performance of LNPs, influencing their biodistribution, cellular uptake, endosomal escape, and ultimately, transfection efficiency and therapeutic efficacy.^[^ ^22^, ^41^, ^42^ ^]^ These lipids typically consist of three components: a hydrophilic headgroup, a linker, and one or more hydrophobic tails. Subtle variations in any of these components can dramatically impact the physicochemical properties and biological performance of LNPs,^[^ ^43^ ^]^ highlighting the significance of fine‐tuning for optimizing therapeutic efficacy. Conventional lipid synthesis strategies, however, are often slow, resource‐intensive, and incompatible with the high‐throughput demands of LNP screening pipelines. These methods typically involve multi‐step synthesis, extensive purification, and solvent exchange procedures, limiting our ability to explore the broad structural diversity of ionizable lipids and speed of LNP discovery.^[^ ^44^ ^]^ Addressing this bottleneck necessitates alternative strategies that streamline reaction procedures while enabling high‐throughput generation of structurally diverse ionizable lipids. Combinatorial chemistry, originally developed in the 1980s for the solid‐phase synthesis of thousands of peptides,^[^ ^45^, ^46^ ^]^ provides a framework for the rapid and systematic synthesis of chemically and structurally diverse molecules. Its application to lipid discovery was demonstrated in 2008 with the generation of the first ionizable lipid library, marking an important step toward high‐throughput exploration of lipid structure‐function relationships.^[^ ^43^ ^]^

**Figure 2:**
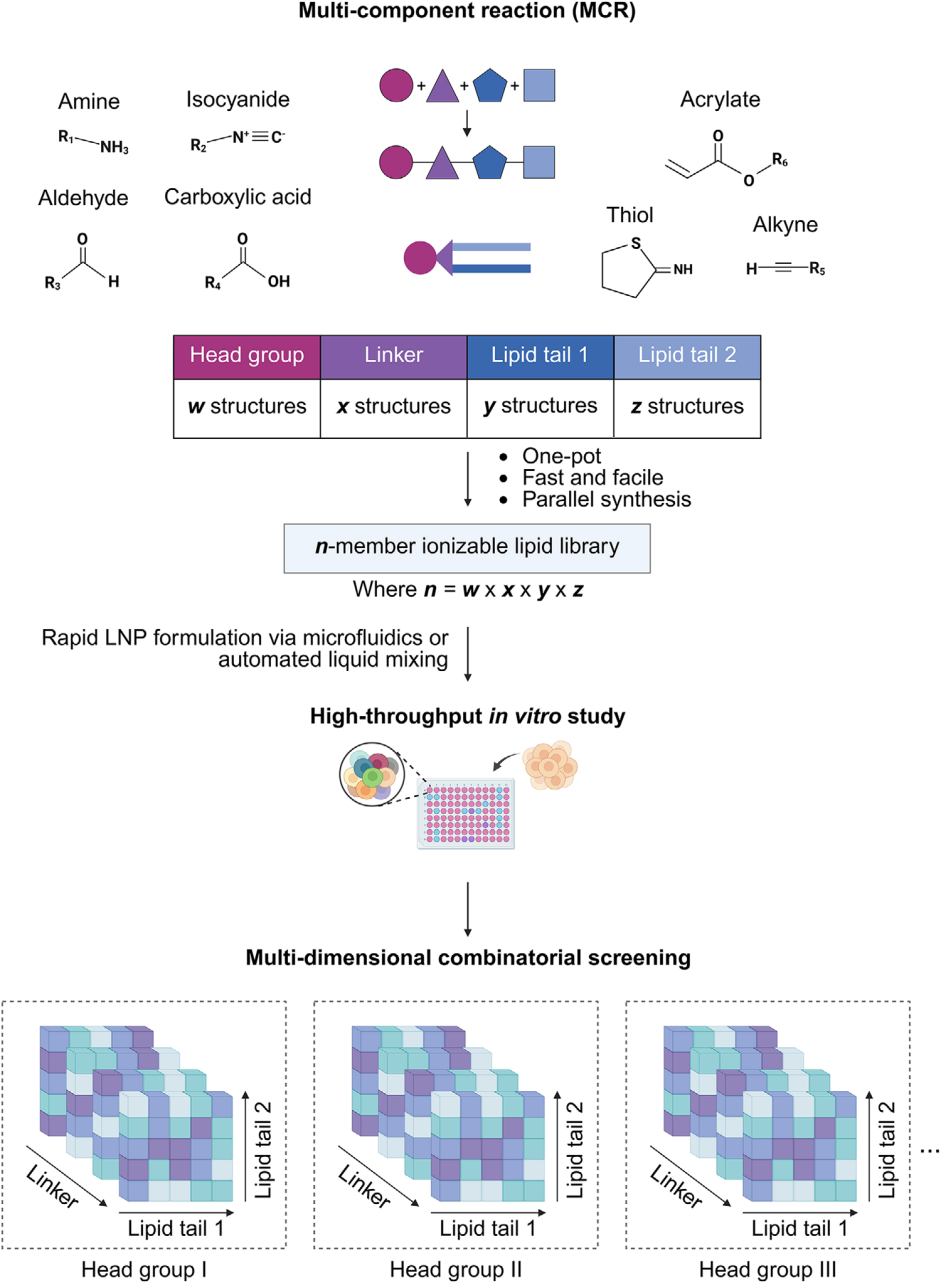
High‐throughput combinatorial synthesis and screening of diverse ionizable lipids via multi‐component reactions (MCRs). MCRs allow the assembly of diverse ionizable lipid structures by combining molecular building blocks (e.g., amines, isocyanides, aldehydes, carboxylic acids, alkynes, acrylates, and thiols). These reactions are typically one‐pot, fast, scalable, and compatible with parallel synthesis in well plate formats. The resulting lipid libraries are formulated into LNPs using microfluidic or automated mixing platforms and evaluated through multi‐parametric screening to identify structural features associated with optimal biological performance. Created with BioRender.

Combinatorial lipid chemistry involves the assembly of molecular “building blocks” into structurally diverse ionizable lipids via a multi‐component reaction (MCR) (**Figure** **2**), in a one‐pot and sometimes catalyst‐free process.^[^ ^44^, ^47^, ^48^ ^]^ In this framework, each chemical component, typically comprising a hydrophilic headgroup, linker, and one or more lipid tails, represents a discrete dimension in the lipid design space. For example, a 4‐component reaction (4‐CR) involving *w* headgroups, *x* linkers, y lipid tail 1 variants, and z lipid tail 2 variants yields a theoretical library size of *n* = *w* × *x* × *y* × *z*, enabling the construction of a chemically diverse, multidimensional lipid library. MCRs are particularly well‐suited for high‐throughput synthesis, as they allow the systematic generation of lipid libraries wherein each variant differs from its neighbors by a single molecular component. This strategy significantly reduces synthetic complexity, enabling parallel synthesis of tens of hundreds of lipids with minimal manual intervention. For instance, a >500‐member library was constructed using a two‐component Michael addition reaction (Michael 2‐CR) between amine‐functionalized headgroups and acrylate‐based hydrophobic tails in a solvent‐ and catalyst‐free manner.^[^ ^44^ ^]^ This reaction produced little to no byproducts, eliminating the need for complex purification and concentration steps. It is not surprising that the Michael addition, broadly characterized as the reaction of an electron‐donating nucleophile with an α, β‐unsaturated carbonyl (double bond linked to a carbonyl group),^[^ ^49^, ^50^ ^]^ has gained popularity in lipid synthesis due to offering benefits such as mild reaction conditions, broad functional group compatibility, and high conversion rates.^[^ ^51^ ^]^ Recently, Li et al., utilized a Michael 3‐CR to combine a nitro ricinoleic acrylate (NRA) linker with aliphatic alcohols (lipid tails) and primary, secondary, or tertiary amines (head groups) to generate a 720‐member ionizable lipid library, enabling the identification of lung‐targeted LNPs for mRNA delivery.^[^ ^52^ ^]^ Despite these advantages, Michael addition reactions have inherent limitations, including prolonged reaction times (ranging from 24 h to 7 days), the requirement for elevated temperatures (90 °C), and structural constraints that often favor symmetric or single‐tailed lipids, features that may limit membrane‐disruptive activity and endosomal escape efficiency.^[^ ^44^, ^53^ ^]^ To address the inherent limitations of Michael addition reaction, alternative combinatorial chemistries have been explored for the synthesis of ionizable lipids.

Among various strategies, the Ugi 3‐component reaction (Ugi 3‐CR) offers a one‐pot approach to generate lipids with asymmetric tails by coupling an amine (headgroup), an isocyanide (lipid tail 1), and an aldehyde or ketone (lipid tail 2).^[^ ^53^ ^]^ This reaction proceeds under mild conditions, yielding structurally diverse products within 12 h and at room temperature. However, it typically requires the use of organic solvents (e.g., methanol) and catalysts.^[^ ^53^, ^54^ ^]^ As an alternative, the Passerini 3‐CR offers similar synthetic versatility while proceeding under catalyst‐free conditions. This reaction combines amine‐containing carboxylic acids (headgroup), isocyanides (lipid tail 1), and aldehydes (lipid tail 2) to produce ester‐ and amide‐containing biodegradable lipids.^[^ ^55^ ^]^ Further dimensionality in combinatorial lipid libraries can be achieved through the Ugi 4‐CR reaction, which introduces a fourth component–a linker molecule into the reaction design. This pathway generates peptide‐like α‐acylaminoamides in high yield and purity via the reaction of amines (headgroup), isocyanides (linker), aldehydes (lipid tail 1), and carboxylic acids (lipid tail 2) in the absence of a catalyst, albeit still requiring a solvent.^[^ ^56^, ^57^ ^]^ Compared to the Ugi 3‐CR, the Ugi 4‐CR requires longer reaction times, multiple steps, and potentially higher temperatures. For example, He et al., developed 161 ionizable lipids by mixing methanol‐dissolved amine and aldehyde for 1 h at room temperature, followed by the addition of carboxylic acid under mixing for a further 30 min, and then the introduction of isocyanide at 45 °C for 12 h.^[^ ^56^ ^]^ Beyond these reactions, several other MCRs have been explored for ionizable lipid synthesis. These include the A^3^ coupling reaction, which combines amines, aldehydes, and alkynes under mild conditions (ethanol as solvent and 50 °C)^[^ ^58^, ^59^ ^]^ and the tandem multi‐component reaction (amine–thiol–acrylate), which enables the rapid (≈1 h), one‐pot synthesis of amidine‐incorporated degradable (AID) lipids with >80% yield via sample sonication at room temperature in the presence of a catalyst.^[^ ^60^ ^]^ It is important to note that, despite significant improvements in throughput compared to conventional lipid synthesis, many of these reactions still require purification to remove byproducts, catalysts, and organic solvents (**Table** **1**).

**Table 1:** Summary of combinatorial synthetic methods used to generate ionizable lipid libraries.

	Lipid Combination	Solvent/Catalyst Requirement	Reaction Temperature	Reaction Time	Post‐Processing
Michael 2‐CR	Amine + Acrylate	None	Elevated (90 °C)	1‐7 days	Not required due to minimal byproducts
Michael 3‐CR	Amine + Acrylate + Aliphatic alcohol	Solvent	Elevated (90 °C)	2‐3 days	Purification
Ugi 3‐CR	Amine + Isocyanide + Aldehyde/Ketone	Solvent and catalyst	Room	12 h	Purification
Ugi 4‐CR	Amine + Isocyanide + Aldehyde/Ketone + carboxylic acid	Solvent	Multiple: room and elevated (45 °C)	14 h	Purification
Passerini 3‐CR	Amine‐containing carboxylic acid + Isocyanide + Aldehyde	Solvent	Room	24 h	Purification
A^3^ coupling	Amine + Aldehyde + Alkyne	Solvent and catalyst	Elevated (50 °C)	8‐48 h	Purification
Tandem MCR	Amine + Thiol + Acrylate	Solvent and catalyst required	Room	1 h	Purification

Although combinatorial lipid chemistry inherently improves the synthetic throughput of ionizable lipid discovery by offering one‐pot simplicity, its full potential is realized only when coupled with high‐throughput experimental platforms. Recent studies have demonstrated the compatibility of MCRs with parallelized synthesis in 96‐well deep‐well plates, where lipid components are mixed in defined molar ratios until reaction completion.^[^ ^53^, ^54^, ^55^ ^]^ These crude products, upon minimal purification, can be directly formulated into LNPs using microfluidic techniques, facilitating rapid downstream evaluation. Recent advancements in automated liquid handling systems have revolutionized this workflow.^[^ ^36^, ^61^ ^]^ For instance, automated liquid handling systems have enabled the synthesis of over 1200 ionizable lipids in a single day, significantly increasing the throughput of lipid synthesis.^[^ ^61^ ^]^ This synergy between combinatorial chemistry and scalable automation allows direct progression from lipid synthesis to LNP formulation and screening, thereby streamlining the entire LNP development pipeline.

## 3 In Vitro High‐Throughput Screening

In the journey toward preclinical development, in vitro high‐throughput screening is an important step in the optimization of LNPs prior to animal studies. Following the generation of combinatorial LNP libraries, efficient in vitro screening workflows are essential for evaluating key biological parameters and prioritizing optimal LNP candidates for in vivo validation. Combinatorial screening, enabled by in vitro HTS, facilitates multidimensional profiling of key parameters such as transfection efficiency, cytotoxicity, cellular uptake, and intracellular trafficking across a range of conditions and cell types. This approach not only accelerates the identification of high‐performing ionizable lipid or LNP formulation from a library of tens to thousands of formulations but also provides mechanistic insights into how physicochemical properties of LNP influence biological performance. By integrating biologically relevant screening strategies early in the development pipeline, in vitro HTS allows streamlining of LNP formulations, reduces reliance on animal models, and shortens optimization cycles, ultimately lowering both cost and development timelines. In this section, we discuss in vitro HTS platforms for LNP screening with a focus on multi‐well plate assays, biomimetic models, and barcoding strategies.

### 3.1 Multi‐Well Plate Cell Culture

A widely adopted approach for in vitro screening of LNPs involves multi‐well plate cell culture assays, which offer parallelized evaluation of their transfection efficiency, cellular uptake, and cytotoxicity. These assays can be conducted in plates with varying well densities, from 6 to 1534 wells, permitting large‐scale formulation screening with high reproducibility and throughput.^[^ ^62^ ^]^ Early LNP screening efforts relied on low‐density multi‐well plate assays and simple cell lines, which, although informative, have limited throughput. To increase the efficiency and scale of LNP screening, combinatorial libraries consisting of diverse LNPs (e.g., varying ionizable lipid structures, helper lipids, cholesterol, and PEG‐lipid ratios) are screened in 96‐well or higher density plates using appropriate culture conditions.^[^ ^52^, ^63^, ^64^, ^65^ ^]^ Assay design in these platforms can be tailored for functional readouts relevant to nucleic acid delivery. For instance, reporter mRNAs (e.g., luciferase) are encapsulated in LNPs and applied to cultured cells, where transfection efficiency is quantified via luminescence or fluorescence‐based assays, providing a quantitative measure of delivery efficiency. Xue et al., screened 180 LNP formulations encapsulating firefly luciferase (Fluc) mRNA in a 96‐well plate setup to rapidly identify formulations with superior transfection efficiency and low cytotoxicity in HeLa cells.^[^ ^64^ ^]^ The successful translation of mRNA into protein was quantified through luminescence assays, demonstrating the use of this platform for rapidly refining large formulation libraries to a small subset of formulations with superior transfection efficiency across various cell types for downstream in vivo validation.^[^ ^66^, ^67^ ^]^ When combined with combinatorial lipid synthesis, these in vitro screening approaches have facilitated multidimensional screening of ionizable lipids to uncover lipid structure‐function relationships crucial for mRNA delivery. For example, systematic variation of lipid tail unsaturation (degree, type, and position) was shown to influence IL‐27 protein expression profiles in the liver in vivo.^[^ ^41^ ^]^ Beyond tail modification, changes in structural features like methylene linker length and headgroup rigidity (linear, cyclic, or aromatic) have been linked to LNP delivery potency in the liver, with some candidates surpassing benchmark ionizable lipids such as SM‐102.^[^ ^55^ ^]^ Notably, incorporation of a benzene ring, as in the 12T‐O14 AID‐lipid, conferred cone‐shaped geometry that enhanced endosomal membrane disruption.^[^ ^60^ ^]^ Moreover, mixing 12T‐O14 with benchmark lipid MC3 in varying ratios allowed tuning of organ‐selective delivery between lung and spleen. Together, these findings reveal how in vitro HTS platforms allow rapid screening of LNP design parameters by mapping LNP structural variations to biological outcomes.

To further improve throughput, researchers have developed combinatorial libraries of ionizable lipids and other components that, when integrated with robotic and microfluidic platforms, allow the rapid synthesis and parallel evaluation of hundreds to thousands of LNP variants under uniform conditions. For instance, Cui et al., reported an automated 384‐well microplate workflow capable of formulating up to 384 distinct LNPs per plate via precise liquid handling, followed by in vitro transfection assays.^[^ ^36^ ^]^ Notably, the top LNP candidates identified by this high‐throughput screening match those from conventional screening, validating the accuracy of the platform. Such automation has enabled the systematic investigation of formulation parameters to map structure‐function relationships and accelerated the iterative design‐build‐test cycle central to LNP development. Overall, multi‐well plate‐based HTS platforms have been used for guiding rational LNP formulation design. These include: i) the relevance of tuning PEG‐lipid content to balance size, stability, and cellular uptake efficiency; ii) the predominant role of ionizable headgroup chemistry in mediating endosomal escape, and iii) the sensitivity of biodistribution to minor compositional changes in LNP. However, the use of simple cell lines and static conditions limits the physiological relevance of these platforms, limiting their predictive accuracy concerning in vivo performance.

### 3.2 Advanced Multi‐Well Cell Culture Models and Organ‐On‐A‐Chip Systems

**Figure 3:**
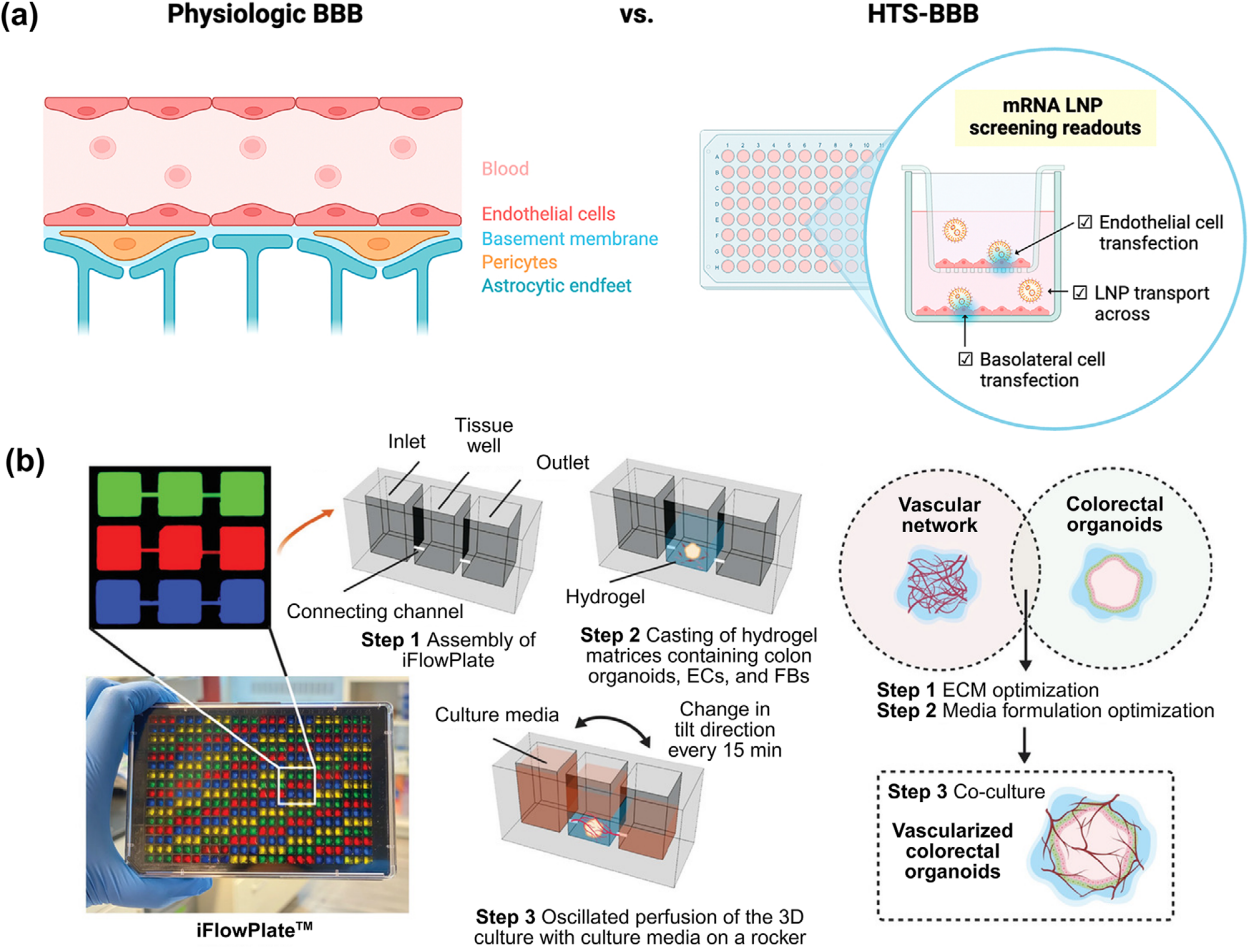
In vitro high‐throughput platforms for screening LNP formulations. a) Schematic of the blood−brain barrier (BBB) physiology and the high‐throughput screening BBB (HTS‐BBB) platform designed to identify LNPs capable of crossing the BBB and transfecting brain endothelial cells. Reprinted with permission from Ref. [70] Copyright 2024, American Chemical Society. b) Illustration of the iFlowPlate system, a high‐throughput screening platform for perfusable 3D tissue models. Left panel: A 384‐well plate is configured with 128 independent units using differently colored dyes for visualization. Middle panel: Each unit contains microchannels connecting the inlet, tissue, and outlet wells, allowing media perfusion across hydrogel matrices containing colon organoids, endothelial cells, and fibroblasts. Right panel: Under dynamic flow conditions, the iFlowPlate facilitates vascularization of cultured colon organoids. Reproduced with permission from Ref. [72] Copyright 2020, Wiley.

To address the limitation of low physiological relevance and enhance the predictive capability of in vitro LNP screening, advanced cell culture models and organ‐on‐a‐chip (OoC) systems have recently emerged as more physiologically relevant alternatives with improved biomimicry of tissue microenvironment and reliability.^[^ ^37^, ^68^, ^69^, ^70^, ^71^ ^]^ One such example is the development of an advanced HTS blood‐brain barrier (BBB) platform incorporating endothelial monolayers with functional tight and adherens junction proteins in a high‐density multi‐well format (**Figure** **3**a).^[^ ^70^ ^]^ While conventional 96‐well brain endothelial monolayers enable HTS, they lack the capacity to measure BBB transport, which requires transwell compartments.^[^ ^70^ ^]^ Transwell systems, typically available in 12‐ or 24‐well formats, can be used to measure transfection efficiency but are impractical for screening large formulation libraries. In contrast, the HTS‐BBB Transwell platform (96‐well format) has been developed to reliably predict LNP transport across the BBB and mRNA transfection in a monolayer of endothelial cells.^[^ ^70^ ^]^ Using this platform, 14 LNP formulations with varying ionizable lipid structures were screened, revealing that ionizable lipid headgroup chemistry had a greater impact on transport performance than LNP size and zeta potential. This highlights the critical role of lipid design in enabling effective delivery to the brain. Furthermore, HTS‐BBB does not directly predict the transfection of deeper brain tissue because the standard model includes only endothelial cells and lacks downstream target brain cell types. However, the predictive accuracy of the model was improved by co‐culturing endothelial monolayers with neurons, suggesting that increasing the complexity of HTS models may enhance physiological relevance for more tailored applications. These findings highlight key principles for brain‐targeted LNP design: i) the role of ionizable lipid chemistry in governing BBB transport; ii) independent assessment of transcytosis and transfection in parallel, and multi‐parametric readouts for refining candidate selection. The HTS‐BBB platform offers a scalable and physiologically relevant approach to systematically map structure–function relationships for tissue‐specific delivery.

Future advancements in developing physiologically relevant models for different applications are expected to further enhance the predictive accuracy of in vitro HTS platforms for LNP screening. One promising example is the use of vascularized microfluidic systems, such as the iFlowPlate (Figure 3b), which was originally developed for vascularized organoid cultures.^[^ ^72^ ^]^ This platform incorporates perfusable endothelial networks and recapitulates dynamic flow conditions, offering a more biomimetic alternative to conventional static assays. The iFlowPlate was shown to maintain stable endothelial monolayers under flow, support vascularized organoid cultures, and enable high content, real‐time imaging across hundreds of wells, demonstrating its capability for physiological relevance and throughput compatibility.^[^ ^72^ ^]^ Although the platform has not yet been applied directly to LNP discovery, its key features, such as fluorescent‐based real‐time tracking, live cell imaging, and perfusable vascular networks that are relevant for evaluating LNP uptake, biodistribution, and transfection across vascular barriers. Nevertheless, realizing the full potential of the iFlowPlate for LNP discovery will require addressing several practical challenges regarding scalability, specifically in terms of fabrication consistency, compatibility with automated liquid handling, and the need for standardized protocols to ensure reproducibility across laboratories. Additionally, endothelial cells within perfusable networks can exhibit sensitivity to flow rate, matrix stiffness, and media composition, all of which may introduce variability in LNP interaction readouts. These limitations must be addressed through standardized protocols and modular assay designs before such platforms can be widely adopted for routine LNP screening. Despite these limitations, the iFlowPlate is a promising physiologically relevant HTS platform that can bridge the gap between traditional static in vitro assays and in vivo models.

Although OoC platforms may be less scalable than conventional platforms and require more complex handling, their ability to simulate human organ environments in vitro (e.g., organ‐specific functions, receptor profiles, and 3D tissue architecture) makes them indispensable for refining lead LNP formulations prior to animal studies. For instance, Moderna researchers integrated the Emulate human Liver‐Chip into their screening workflow to identify LNPs for treating liver fibrosis.^[^ ^73^, ^74^ ^]^ Traditional animal models often fail to predict human‐specific toxicity, particularly with novel LNP chemistries. The Liver‐Chip enabled early detection of gene signatures correlated with adverse outcomes in non‐human primates, as well as collagen remodeling indicative of pro‐fibrotic responses. Critically, it also distinguished between LNP‐ and mRNA‐mediated toxicity and uncovered mechanistic pathways such as necroptosis. Over 18 months, the team screened 35 LNP formulations for approximately USD 325K, over four times faster and at a fraction of the cost of equivalent non‐human primate studies, which would have required more than USD 5 M and taken over five years. These results highlight the potential of humanized OoC platforms to deliver human‐relevant data at a throughput and cost that make them suitable for screening LNP candidates before advancing to in vivo evaluation. Therefore, future development should focus on improving the scalability and automation of such platforms to enable their integration into parallelized HTS workflows, thereby enhancing physiological relevance, reducing dependence on animal models with limited predictive capability for human outcomes, and accelerating decision‐making for downstream in vivo screening.

### 3.3 NanoPRISM

**Figure 4:**
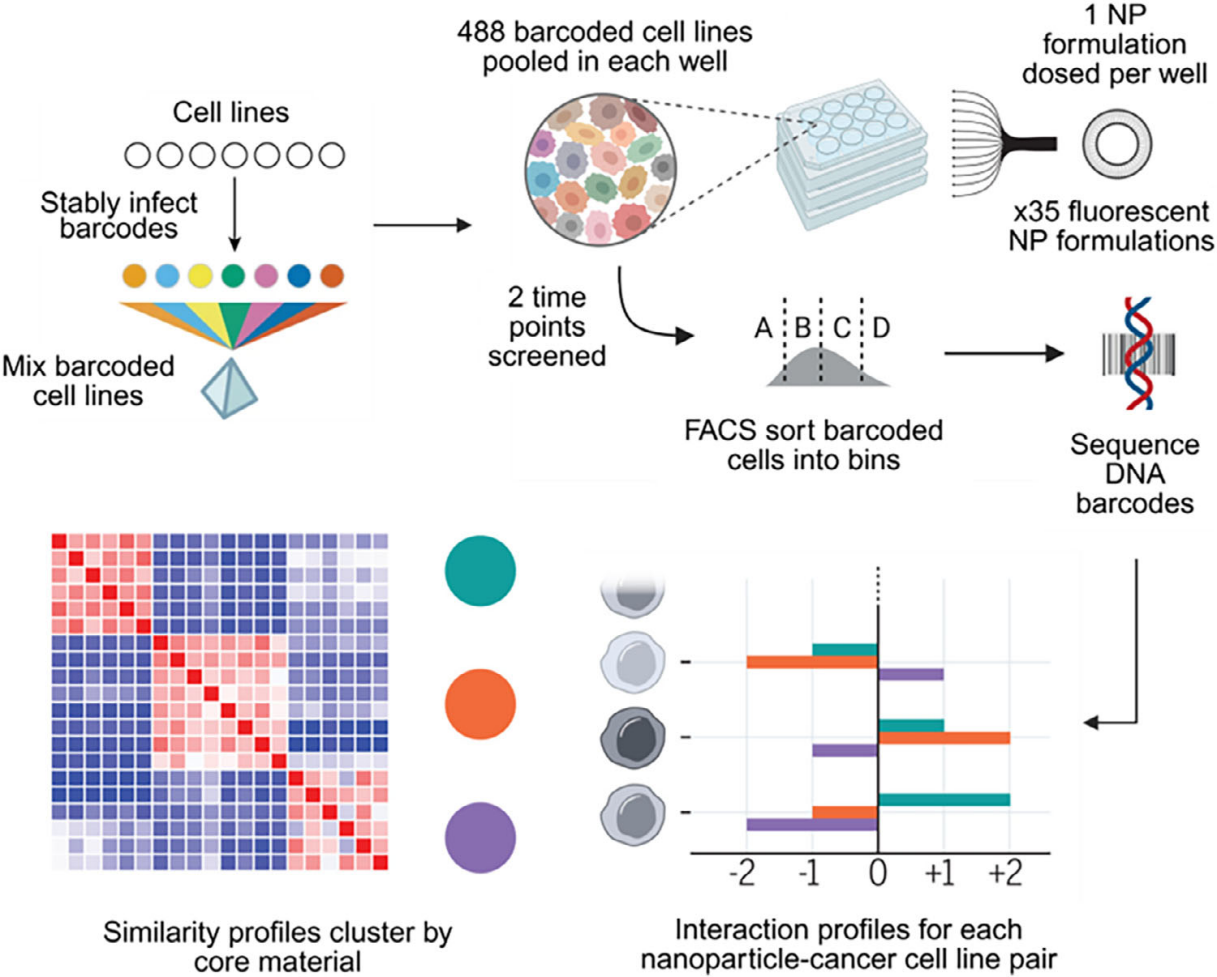
High‐throughput screening of NP‐cell interactions using the nanoPRISM platform. A pooled library of 488 barcoded cancer cell lines is exposed to individual fluorescently labeled NPs. After incubation, cells are sorted by fluorescence intensity into four bins via fluorescence‐activated cell sorting (FACS), showing differential NP uptake. DNA barcodes from each bin are extracted and sequenced to quantify the distribution of individual cell lines across uptake levels. This allows the construction of interaction profiles for each NP‐cell line pair and reveals clustering of responses based on core materials (NPs). The top left image was adapted with permission from Ref. [75] Copyright 2016, Springer Nature. Other images were adapted with permission from Ref. [78] Copyright 2022, American Association for the Advancement of Science (AAAS).

The nanoPRISM method is a multiplexed screening method that combines nucleic acid barcoding with multi‐well plate‐based LNP screening. This technique is based on the PRISM method, which has previously been used to reliably screen a massive library of existing and potential anticancer drugs across multiple cancer cell lines.^[^ ^75^, ^76^, ^77^ ^]^ The nanoPRISM method uses nucleic acid barcoding to simultaneously screen NP interaction with different cell types in a single experiment. Specifically, lentiviral barcoding plasmids containing unique 24‐base pair DNA sequences (barcodes) are transfected into host cells (e.g., HEK‐293T). The barcoded cells are pooled together in equal proportions, seeded into multi‐well plates, and subsequently exposed to various NP formulations, including multiple liposomal variants. Using this strategy, Boehnke et al., investigated the interactions of 35 distinct NP formulations, including various liposomal NPs, across 488 cancer cell lines (**Figure** **4**).^[^ ^78^ ^]^ The screening process involved the following steps: 1) transfecting each cancer cell line with a plasmid containing a unique oligonucleotide barcode, 2) pooling the barcoded cells in equal proportions, 3) seeding the pooled cells on microplates, with each well treated with a different NP formulation, 4) sorting cells based on their fluorescence intensity using fluorescence‐activated cell sorting (FACS), and 5) extracting, amplifying, sequencing, and analyzing the DNA barcodes to determine NP uptake and response across cell types. Their methodology demonstrated that nucleic acid barcoding significantly enhanced screening throughout, overcoming the limitations of conventional methods, which typically assess only a small subset of formulations per experiment. Notably, this method allows the rapid, high‐throughput assessment of NP‐cell interactions at an unprecedented scale, revealing critical formulation‐dependent effects on cellular uptake and providing robust data to inform formulation optimization.

The nanoPRISM platform, when integrated with multiomics and ML approaches, enables high‐throughput profiling of NP‐cell interactions across a pool of 488 different cancer cell lines. This large‐scale screening revealed that the NP core materials (liposome, PLGA, or polystyrene) were the primary determinant of cell‐type‐specific association rather than the influence of surface chemistry. A comparative screening of liposomes with different surface chemistries showed that natural polysaccharide coatings increased receptor‐mediated interactions, while PEGylation broadly reduced both specific and non‐specific binding. By linking NP uptake profiles with multiomics data using a random forest model across 488 cell lines, the study identified SLC46A3 as a negative regulator of both liposomal and LNP uptake. Notably, cell lines with low SLC46A3 expression exhibited markedly higher cellular uptake and transfection efficiency. These findings were validated using GFP were experimentally validated using GFP‐encoding LNPs, establishing a mechanistic link between cellular genotype and functional delivery. This study demonstrates the power of integrating nanoPRISM with multiomic analysis to uncover predictive biomarkers and design rules that would remain inaccessible through conventional low‐throughput screening. Despite its advantages, nanoPRISM has limitations. It primarily assesses cellular association or NP uptake rather than intracellular payload delivery or therapeutic efficacy. Cellular internalization alone does not guarantee biological activity or therapeutic benefit, potentially limiting the predictive accuracy of this screening method. Furthermore, variations in barcoding efficiency across different cell lines could introduce biases, requiring careful interpretation of the obtained data. This method is restricted to in vitro conditions, thus neglecting the complex in vivo tissue microenvironment. Future improvements could involve integrating barcoding approaches that can measure therapeutic payload and intracellular delivery while mimicking physiologically relevant in vitro models to improve the predictive accuracy of nanoPRISM.

## 4 In Vivo High‐Throughput Screening

In vitro models often fall short in predicting NP efficacy in vivo due to their inability to fully replicate physiological and pathological complexities.^[^ ^21^, ^68^, ^69^, ^79^, ^80^ ^]^ Biological barriers such as protein corona formation upon systematic administration, clearance by the mononuclear phagocyte system, and tissue‐specific endothelial barriers (e.g., in liver, lung, or brain) can dramatically alter NP behavior in vivo, factors not adequately captured in static 2D cultures or even advanced organ‐on‐chip platforms.^[^ ^81^, ^82^ ^]^ As a result, some LNPs that perform well in vitro may fail in vivo, and vice versa. For instance, Hamilton et al., demonstrated that ionizable lipids with polyamine cores 482, 488, 494, and c494 transfected immune cells efficiently in vivo, even though the 482 core had previously exhibited poor in vitro performance.^[^ ^83^ ^]^ Thus, multiplexed in vivo screening is essential to avoid false negatives. Although advanced in vitro models such as the HTS‐BBB and OoC platforms can improve predictive accuracy, in vivo assessment remains the most physiologically relevant approach to streamlining LNP formulations. Conventional in vivo screening approaches, such as intravenous administration followed by imaging and histology, are inherently low‐throughput and require a large number of animals to screen multiple LNP formulations, making large‐scale studies impractical due to the high costs and ethical concerns associated with animal use. To address these limitations, recent advances in multiplexed screening techniques, particularly nucleic acid and peptide barcoding, have enabled simultaneous assessment of tens to hundreds of LNP formulations within a single animal, reducing animal use and increasing screening efficiency, offering a scalable strategy for directly linking LNP structure‐function relationships in vivo.

### 4.1 Nucleic Acid Barcoding

**Figure 5:**
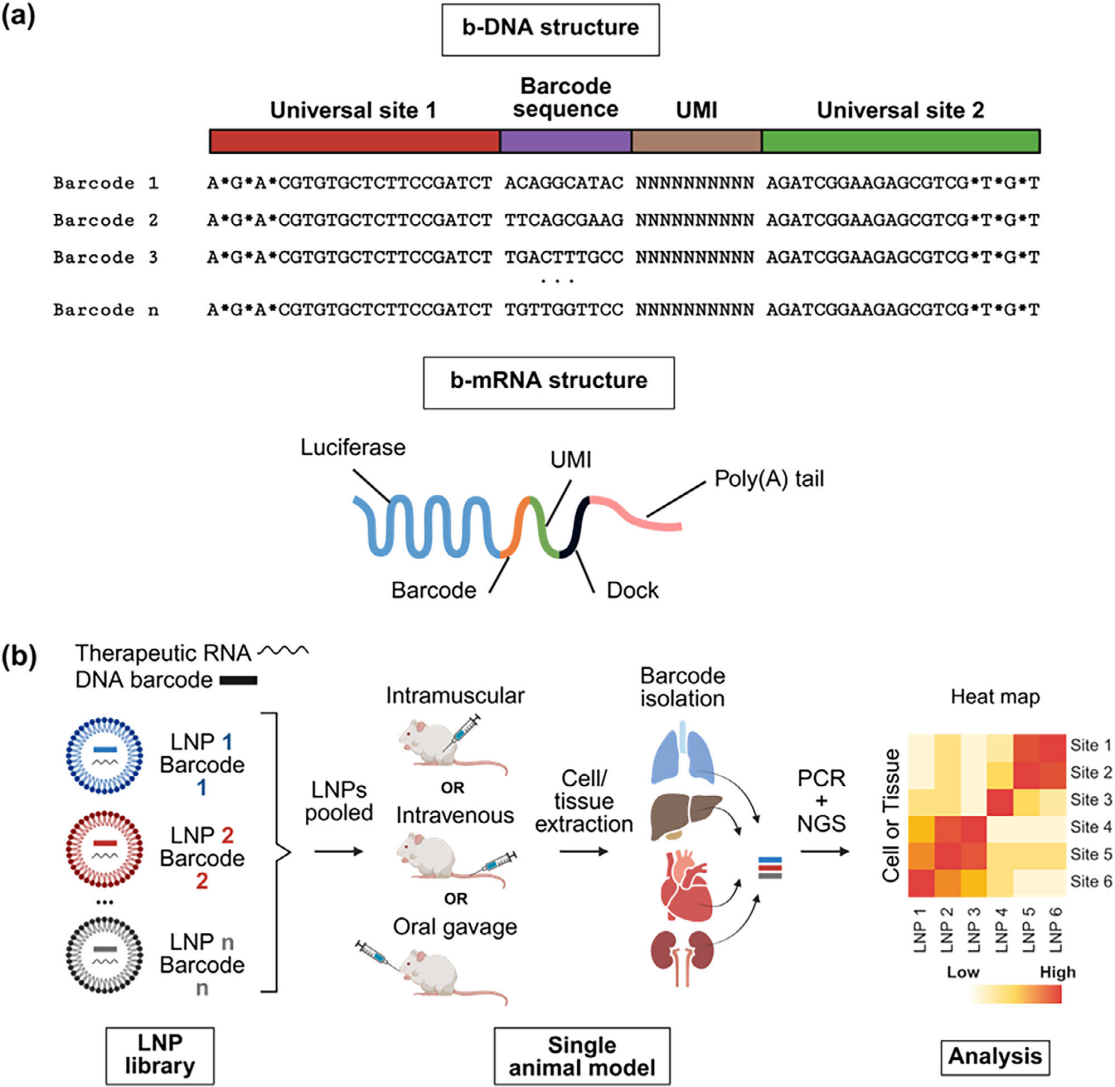
Nucleic acid barcoding strategies for high‐throughput in vivo screening of LNP libraries. a) Design of barcoded DNA (b‐DNA) and barcoded mRNA (b‐mRNA) sequences used for LNP tracking. b‐DNA molecules incorporate universal priming sites, a unique barcode sequence, and a unique molecular identifier (UMI), with phosphorothioate (*) modifications enhancing nuclease resistance. Example sequence adapted with permission from Ref. [85] Copyright 2017, National Academy of Sciences. b‐mRNA incorporates a barcode, UMI, and docking region fused to a luciferase reporter and poly(A) tail, allowing transcript‐based tracking. Adapted with permission from Ref. [86] Copyright 2019, Elsevier. b) Each LNP formulation in an n‐member library is labeled with a unique b‐DNA or b‐RNA sequence. Pooled LNPs are administered into a single animal model via different routes. After cells or tissue isolation, barcodes are recovered and quantified using NGS. Heat maps generated from sequencing data report the normalized distribution of each LNP across cell and tissue types, enabling quantitative comparison of delivery efficiency. Schematic created with BioRender.

Nucleic acid barcoding combined with next‐generation sequencing (NGS) allows a high‐throughput multiplexed evaluation of LNP delivery within a single animal^[^ ^21^, ^36^, ^64^, ^79^, ^80^, ^83^, ^84^, ^85^, ^86^, ^87^, ^88^ ^]^ (**Figure** **5**b). By sequencing unique barcodes associated with each LNP formulation, NGS allows the parallel analysis of multiple formulations in a single experiment, significantly enhancing screening efficiency. This high‐throughput technique follows four key steps: 1) LNPs are formulated with barcoded DNA (b‐DNA) or mRNA (b‐mRNA) using microfluidic mixing, assigning each formulation a unique nucleic acid identifier; 2) pooled LNPs are administrated into a single animal at a fixed nucleic acid dose; 3) at predetermined time points, target tissues or cells are collected, barcode sequences are extracted and polymerase chain reaction (PCR) amplification is performed to ensure sufficient quantity of barcode sequences for analysis; and 4) deep sequencing with NGS to quantify LNP distribution in different tissues (Figure 5b). The sequencing data are normalized across different tissues within the same animal, providing a relative quantification of LNP accumulations in the cells or tissues of interest.^[^ ^79^, ^85^, ^87^ ^]^ For example, if the sequencing reads for barcode 1, barcode 2, and barcode 3 in a given cell type are 5000, 3000, and 2000, the normalized deliveries of LNP 1, LNP 2, and LNP 3 are 50%, 30%, and 20%, respectively.

**Figure 6:**
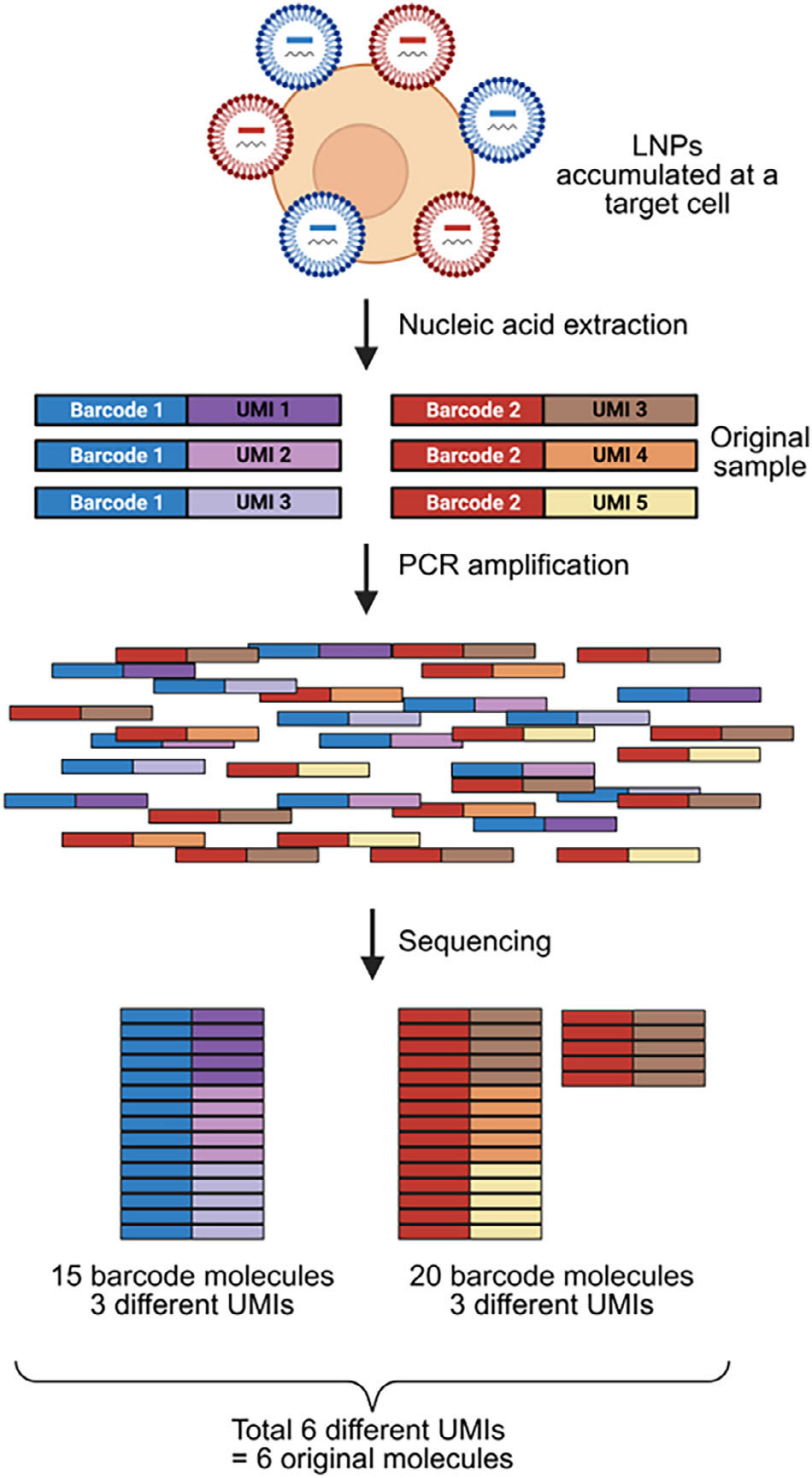
A simplified example of UMI's role in NGS. Two LNP formulations accumulate at a target cell and release barcoded nucleic acid, which is subsequently extracted for analysis. Although the original sample contains an equal number of barcodes 1 and 2 molecules, downstream PCR amplification introduces bias, leading to overrepresentation of barcode 2 sequences during sequencing. By incorporating UMIs into each barcode molecule prior to amplification, the number of unique original molecules can be accurately determined, independent of amplification bias. In this example, despite differences in sequencing read counts, UMI deduplication reveals that only six unique molecules (based on six distinct UMIs) were present in the original sample. Schematic created with BioRender.

Nucleic acid barcodes can be subdivided into different segments (Figure 5a). b‐DNA consists of two universal primer binding sites, a barcode region unique to each LNP composition, and a unique molecular identifier (UMI). The universal sites, shared across all barcodes, serve as primer binding sites for initiating PCR amplification^[^ ^64^, ^85^, ^86^ ^]^ and undergo phosphorothioate modifications to increase barcode stability and decrease exonuclease degradation.^[^ ^79^, ^85^ ^]^ The barcode region ensures that all LNPs with the same composition carry the same barcode (Figure 5b). For instance, LNPs with composition 1 contain barcode sequence 1, LNPs with composition 2 contain barcode sequence 2, and so on. UMIs, in contrast, are randomly generated sequences incorporated into each barcode molecule so that each molecule can be uniquely identified.^[^ ^79^, ^83^, ^86^, ^89^ ^]^ This allows one to distinguish true molecule counts from PCR amplification artifacts and correct for unequal amplification across barcodes. Because each UMI is counted only once regardless of amplification, the absolute number of original barcodes is preserved (**Figure** **6**).^[^ ^90^ ^]^ This prevents overrepresentation of barcode sequences from PCR bias, enabling more accurate quantification of LNP distribution.

 b‐mRNA has a more complex design with five segments: a functional mRNA region, a barcode region, a UMI, a PCR docking site, and a long chain of adenosine called the poly(A) tail (Figure 5a).^[^ ^86^ ^]^ The functional mRNA sequence is translated into protein, while the barcode tracks delivery, and UMI controls PCR amplification bias. The PCR docking site acts as a universal primer‐binding site for PCR amplification.^[^ ^91^ ^]^ The poly(A) tail supports mRNA translation and improves its stability by restricting exonuclease access, and provides a binding site for reverse transcriptase to generate complementary DNA (cDNA) for subsequent PCR amplification.^[^ ^86^, ^92^ ^]^

Nucleic acid barcoding‐based in vivo HTS approach offers significant advantages over conventional in vivo screening. First, multiplexed screening reduces the number of animals required to screen hundreds of formulations compared to the larger animal cohorts required in low‐throughput techniques, screening only a few LNPs at a time. Second, PCR amplification enables high‐sensitivity detection of nucleic acid doses as low as 0.0001 mg kg^−1^,^[^ ^85^ ^]^ minimizing the amount of LNPs required for screening, thereby reducing both time and cost. Conventional in vivo studies, by comparison, have lower limits of detection and require larger amounts of LNPs per formulation.

In recent years, nucleic acid barcoding has led to important insights into the relationship between LNP design and site‐specific accumulation. For example, subtle modifications in LNP composition, ranging from changes in individual constituents to subtle molecular‐level alterations, influence their biodistribution and delivery efficiency.^[^ ^21^, ^36^, ^64^, ^79^, ^80^, ^83^, ^84^, ^85^, ^87^, ^88^ ^]^ While it is widely assumed that changes in LNP composition influence their size and hence delivery, in vivo HTS studies have revealed no correlation between LNP size and delivery efficacy.^[^ ^87^, ^88^ ^]^ This finding suggests that size may not be a confounding factor for LNP delivery, reinforcing the validity of observed structure‐function relationships. Traditionally, ligand conjugation has been used for selective targeting. Strong evidence suggests that lipid structure and LNP composition alone can influence tissue‐specific accumulation without targeting ligands.^[^ ^93^, ^94^, ^95^, ^96^, ^97^ ^]^ The integration of nucleic acid barcoding and NGS has enabled rapid identification of several key parameters affecting site‐specific LNP accumulation in a high‐throughput manner.^[^ ^65^, ^80^, ^84^, ^85^, ^88^ ^]^ For example, LNP biodistribution is significantly affected by the administration route,^[^ ^83^ ^]^ ionizable lipid,^[^ ^64^, ^83^, ^88^ ^]^ PEG‐lipid,^[^ ^21^, ^64^, ^79^, ^84^, ^88^ ^]^ helper lipid,^[^ ^79^ ^]^ cholesterol,^[^ ^87^ ^]^ and constituent molar ratios.^[^ ^64^, ^79^, ^83^, ^85^, ^88^ ^]^ A remarkable discovery is that molecular‐level modifications to ionizable lipids, such as changing the amine head of an ionizable lipid, appear to significantly impact biodistribution.^[^ ^64^ ^]^ These promising results highlight the potential of nucleic acid barcoding to systematically identify key determinants of LNP delivery, establishing the rational design rules for efficient delivery systems.

Beyond identifying LNP biodistribution, nucleic acid barcoding can also uncover the molecular pathways that govern LNP tropism. The protein corona itself can be leveraged to control LNP organ tropism,^[^ ^98^, ^99^ ^]^ a property that can be tuned through LNP composition modifications.^[^ ^93^, ^100^, ^101^, ^102^ ^]^ For example, certain LNPs have been proposed to enter hepatocytes via apolipoprotein E (ApoE) and low‐density lipoprotein receptor (LDLR) mediated endocytosis.^[^ ^103^ ^]^ However, alternative pathways have also been identified; serum albumin, for instance, can facilitate LNP uptake via an ApoE‐independent pathway.^[^ ^104^ ^]^ A study investigating the LAMP1 gene silencing by siRNA‐carrying LNPs in wild‐type as well as ApoE, LDLR, and very low‐density lipoprotein receptor (VLDLR) knockout mice showed that LNPs can achieve non‐hepatocyte tropism through a mechanism independent of LDLR, VLDLR, or APoE.^[^ ^88^ ^]^ These findings demonstrate the effectiveness of nucleic acid barcoding in elucidating the molecular mechanism driving LNP tropism across diverse formulations.

Recently, the FIND (Fast Identification of Nanoparticle Delivery) was developed by combining DNA barcoding with Cre mRNA to assess functional mRNA delivery in addition to biodistribution.^[^ ^79^ ^]^ In this approach, successfully transfected Ai14 cells undergo Cre‐mediated recombination, triggering robust tdTomato fluorescence that colocalizes with barcoded DNA to quantify functional delivery.^[^ ^79^, ^106^ ^]^ Although FIND measures functional delivery more accurately than DNA barcoding alone, it cannot identify which specific LNP mediates mRNA translation in a given cell. When multiple LNPs enter the same cell, any one of them (or several in combination) may be responsible for the observed fluorescence. mRNA barcoding addresses this by linking barcode identity to the translatable mRNA sequence while maintaining structural similarity to native mRNA,^[^ ^86^ ^]^ potentially predicting delivery more accurately. Yet, successful delivery of barcoded mRNA does not guarantee its translation into functional protein, and this approach is not suitable for gene silencing or knockdown efficiency. Furthermore, FIND only reports tissue‐level data from the bulk‐sorted tdTomato+ cells, capturing cell‐type‐level differences but not heterogeneity within those populations. Other limitations include fluorescent signal saturation and reliance on the transgenic Ai14 mouse model, restricting its applicability in larger animal or disease models where Cre‐Lox reporters are not available.^[^ ^79^, ^105^ ^]^

To overcome the limitations of transgenic Cre‐Lox systems and improve translational relevance, more versatile platforms such as SANDS (Species‐Agnostic Nanoparticle Delivery Screening) and SENT‐seq (Single‐cell Nanoparticle Targeting‐sequencing) have been developed. Traditional in vivo HTS relies on rodent models, which are genetically and physiologically distinct from primates, leading to species‐dependent differences in LNP biodistribution and cellular uptake that limit the predictability for non‐human primates (NHPs) and humans.^[^ ^107^, ^108^, ^109^, ^110^ ^]^ The SANDS platform addresses this gap by evaluating functional LNP delivery across humanized, primatized, and murinized liver types within the same animal model, without requiring transgenic fluorescence reporters.^[^ ^107^ ^]^ In this approach, each LNP co‐delivers both b‐DNA and mRNA encoding glycosylphosphatidylinositol (GPI)‐anchored camelid single‐domain antibody (referred to as aVHH). Following pooled LNP administration into mice engrafted with human, NHP, or native hepatocytes, aVHH+ cells are isolated via FACS (using a fluorescently tagged anti‐VHH antibody that binds aVHH expressed on the cell surface). Sequencing the barcodes in these cells reveals species‐dependent differences in functional delivery between human, primate, or mouse hepatocytes. SANDS data have shown that rodent models often overestimate or misrepresent LNP efficacy in primates and humans, likely due to endocytic and mRNA translation pathways. This platform also highlighted that PEG‐lipid tail length and ionizable lipid type affect delivery in a species‐specific manner, emphasizing the need for tailored LNP design criteria. While humanized animal models have become more widespread for LNP screening,^[^ ^111^, ^112^, ^113^, ^114^ ^]^ their integration into high‐throughput workflows remains limited. Furthermore, like FIND, SANDS reports tissue‐level data only with limited cellular resolution.

The SENT‐seq platform goes a step further by achieving single‐cell resolution without the need for transgenic mouse models. Like SANDS, each LNP carries both b‐DNA and aVHH‐encoding mRNA. After pooled administration, the liver is harvested and dissociated into a single aVHH+ cell in suspension. Rather than requiring a transgenic fluorescent protein, SENT‐seq uses magnetic polymer beads conjugated to oligonucleotides with two orthogonal capture sequences: one for b‐DNA, and another for endogenous mRNA and DNA‐tagged anti‐aVHH antibodies.^[^ ^115^ ^]^ These beads simultaneously label each aVHH‐expressing cell, capture the co‐delivered b‐DNA, and record a sample of the cell's mRNA transcriptome. Subsequently, single‐cell RNA sequencing (scRNA‐seq) provides three readouts: i) each cell identity and state from endogenous mRNA profiles, ii) functional delivery from DNA‐tagged antibody readouts, and iii) evidence of mRNA translation from detection of the aVHH protein. Using SENT‐seq, Dobrowolski et al., identified liver cell subpopulations with high or low LNP uptake and linked cell‐intrinsic factors, such as CDK13 and CDK14 expression, to effective mRNA delivery and translation.^[^ ^115^ ^]^ More importantly, its application to NHPs showed that LNPs performing well in mice often exhibit distinct biodistribution and delivery profiles in primate cells,^[^ ^110^ ^]^ highlighting the relevance of cross‐species functional datasets to guide clinical translation.

Complementary to single‐cell sequencing, spatial transcriptomics (ST) provides microenvironment‐level resolution for mapping LNP delivery within intact tissues. By preserving tissue architecture while profiling gene expression in situ, ST can reveal which cell types and regions within organs receive functional delivery. For example, a recent high‐resolution in situ transcriptomic analysis showed that intravenous LNP‐mediated mRNA delivery in the brain occurred predominantly in endothelial cells, especially those adjacent to vascular smooth muscle cells in blood vessels.^[^ ^116^ ^]^ This suggests that local microenvironment features (such as proximity to vasculature) can strongly influence LNP uptake. Combined with barcoding, ST can map where functional delivery events happen within the tissue, directly linking LNP performance to specific anatomical regions. In hepatocellular carcinoma models, ST and scRNA‐seq together revealed how siRNA‐loaded LNPs reprogrammed the specific tumor region, activating immune interferon signaling and promoting immune infiltration.^[^ ^117^ ^]^ As single‐cell and ST approaches gain wider adoption, they are reshaping how targeting specificity is evaluated. ST in particular can complement barcoding techniques such as SANDS and SET‐seq by resolving LNP delivery at single‐cell and sub‐tissue resolution that goes beyond traditional tissue‐level biodistribution readouts.

The choice of nucleic acid (e.g., siRNA or mRNA) cargo itself also influences the size and structure of LNPs as well as the spatial distribution of various components, such as cholesterol and helper lipids,^[^ ^118^, ^119^ ^]^ thereby affecting their in vivo delivery. Guimaraes et al., demonstrated that replacing b‐mRNA with b‐DNA resulted in changes in LNP hydrodynamic diameter and polydispersity index (PDI), which in turn influenced in vivo delivery efficiency.^[^ ^86^ ^]^ Given that b‐DNA is orders of magnitude smaller than mRNA, this size discrepancy likely contributed to changes in LNP size. However, this study assessed the delivery of b‐DNA and b‐mRNA separately but not the codelivery of b‐DNA and functional mRNA. In contrast, another study evaluating co‐delivery of functional mRNA and b‐DNA found that incorporating b‐DNA did not significantly alter LNP properties.^[^ ^64^ ^]^ This suggests that the presence of a smaller b‐DNA alongside a larger mRNA does not drastically affect LNP size. Furthermore, a strong correlation was observed between b‐DNA and siRNA delivery, where LNPs with low b‐DNA accumulation in the liver also displayed lower gene silencing efficiency with siRNA.^[^ ^85^ ^]^ This may be due to the comparable sizes of b‐DNA and siRNA,^[^ ^86^ ^]^ minimizing cargo‐induced alterations in LNP size. However, the co‐delivery of b‐DNA and siRNA could still influence LNP biodistribution. To date, only one study has assessed the effects of b‐mRNA. Further work is required to determine the effects of b‐DNA vs b‐mRNA on LNP properties and in vivo fate. More importantly, the choice of nucleic acid barcode could be critical in predicting LNP delivery efficiency. While the size of b‐DNA is comparable to siRNA and sgRNA, it is substantially smaller than mRNA.^[^ ^86^ ^]^ Thus, choosing a barcode with minimal influence on LNP properties will be essential for improving the accuracy of delivery efficacy predictions.

**Figure 7:**
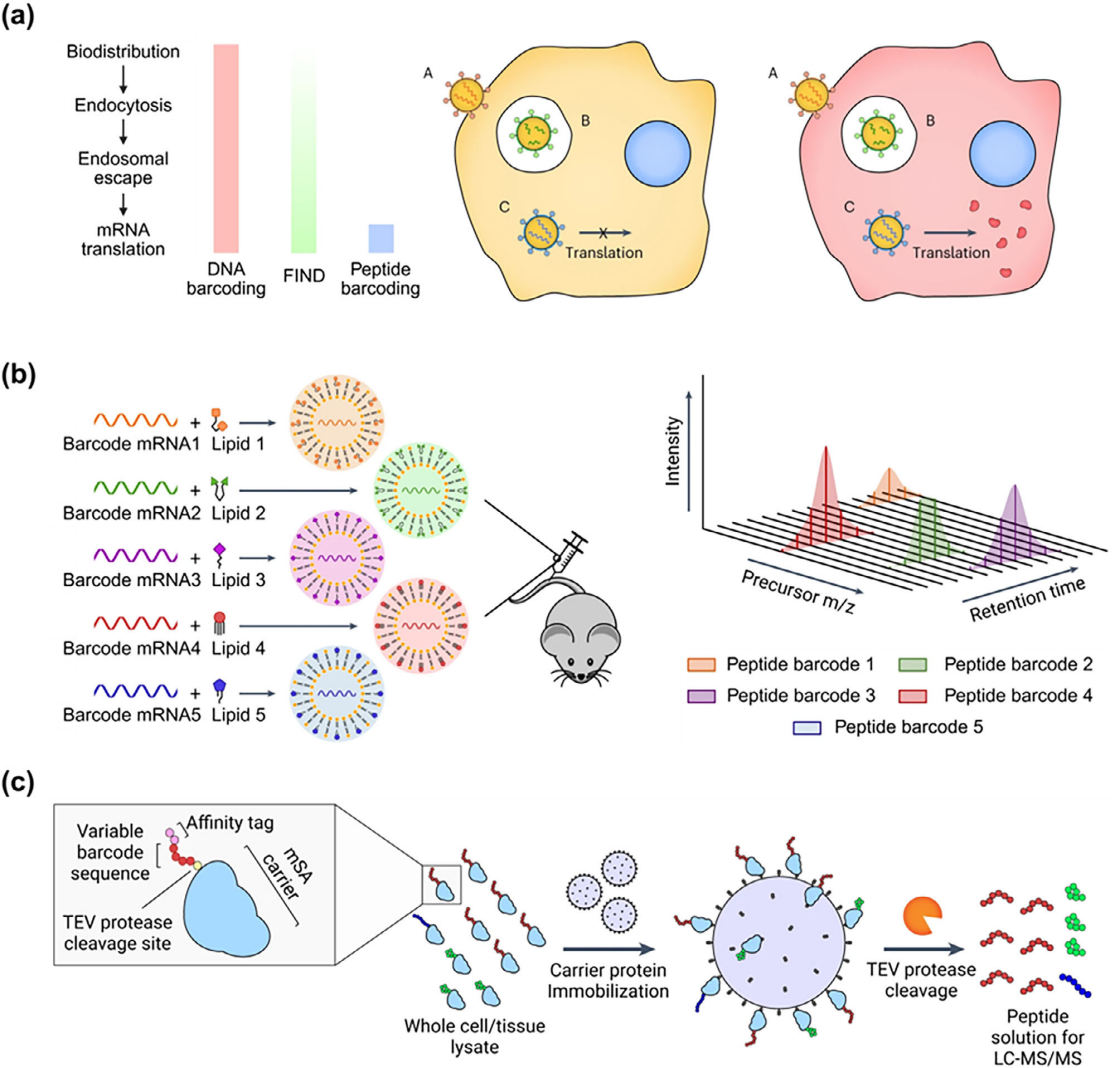
Peptide barcoding strategy for in vivo screening of mRNA LNP formulations. a) Comparison of barcoding strategies based on their resolution in detecting different stages of intracellular delivery. DNA barcoding tracks all LNPs that reach the target tissue, FIND identifies those that escape endosomes, while peptide barcoding selectively identifies LNPs that successfully deliver mRNA, leading to translation. In this example, LNP B and C are internalized, but only B enables cytosolic mRNA release and protein expression; peptide barcoding captures only this productive delivery event. LNP A remained on the cell surface without internalization. b) A library of LNPs is formulated with a unique peptide barcode mRNA and pooled for systemic administration into mice (left). LC‐MS/MS output shows the detection of peptide barcodes based on their precursor mass‐to‐charge ratio (m/z), retention time, and signal intensity (right). Each peak corresponds to a distinct LNP formulation that successfully delivered and translated mRNA in vivo. c) Upon successful delivery and translation, each mRNA expresses a carrier protein fused to a unique peptide barcode. Following tissue lysis, barcoded peptides are isolated via affinity enrichment, enzymatically cleaved, and quantified by LC‐MS/MS to determine the relative efficacy of each LNP formulation. Adapted with permission from Ref. [105] Copyright 2023, Springer Nature.

DNA and mRNA barcoding present several key limitations in assessing LNP delivery (**Table** **2**). A major challenge of DNA and mRNA barcoding is their inability to differentiate between LNPs that have functionally delivered a therapeutic payload, those that remain on the cell surface, and those internalized but lack functional activity (**Figure** **7**a). FIND, SANDS, and SENT‐seq cannot definitively attribute a functional delivery event to a single LNP when many formulations are tested in parallel. While mRNA barcoding may provide a more accurate measure of functional delivery compared to FIND or other barcoding methods, its validation has been limited to a small number of LNP formulations.^[^ ^86^ ^]^ This raises the possibility that certain LNP formulations may accumulate at a target site without being translated into b‐mRNA. Ultimately, LNP efficacy should be evaluated based on both biodistribution and functional delivery rather than each factor separately. Given the inherent size differences between b‐DNA, b‐mRNA, siRNA, and sgRNA, the barcode type must be carefully selected according to the application to minimize unintended effects on LNP properties. Furthermore, some degree of bias from PCR or sequencing is inevitable. More broadly, these barcoding strategies apply only to NPs compatible with nucleic acid delivery, as they must accommodate barcodes without altering their properties. For NPs incompatible with nucleic acid delivery, alternative high‐throughput screening methods are required.

**Table 2:** Comparison of in vivo barcoding strategies for LNP screening.

	DNA Barcoding	mRNA Barcoding	Peptide Barcode
Quantification method	Next‐generation sequencing	Next‐generation sequencing	Liquid chromatography and tandem mass spectrometry
Sensitivity	High (0.1 µg kg^−1^)	High (0.1 µg kg^−1^)	Potentially lower (1 µg kg^−1^)
Applications	Gene silencing or knockdown (siRNA, sgRNA), protein expression (mRNA)	Protein expression (mRNA)	Protein expression (mRNA)
Functional delivery	Potentially least accurate	Potentially accurate	Most accurate
Multiplexed screening	Yes	Yes	Yes
General Limitations	PCR bias Sequencing bias	PCR bias Sequencing bias	Biodistribution cannot be assessed without RNA sequencing or other tracking methods
	The translation efficiency of exogenous mRNA differs between cell types		
	LNP pooling may introduce non‐linear effects that deviate from analyses of single LNP species		
	Only compatible with NPs capable of nucleic acid delivery		

### 4.2 Peptide Barcoding

A peptide barcoding platform encoded by mRNA is a high‐throughput screening strategy to assess functional LNP delivery by tracking mRNA translation into proteins rather than just measuring mRNA biodistribution. Unlike nucleic acid barcoding, which identifies where LNPs accumulate, peptide barcoding provides a direct readout of functional protein expression in target cells (Figure 7a).^[^ ^105^ ^]^ This approach overcomes a major limitation of FIND and other nucleic acid barcoding strategies, which detect LNP presence but do not confirm whether mRNA translation has occurred. In this approach, each LNP is barcoded with an mRNA encoding a unique peptide tag. Upon successful delivery and translation, this mRNA produces a distinct peptide sequence that serves as a marker for functional delivery. The relative abundance of each peptide barcode is then quantified using liquid chromatography followed by tandem mass spectrometry (LC‐MS/MS).

To design the platform, LNPs are formulated with mRNA‐encoding peptide barcode proteins. These proteins consist of two components: a carrier protein, monomeric streptavidin to reduce background noise from miscellaneous proteins, and a variable barcode sequence with an affinity tag fused to the carrier protein through a TEV protease cleavage site (Figure 7c). Similar to the previous nucleic acid barcoding method, the barcoded LNPs are pooled and administered to a single animal. After extracting and processing tissues, lysates are produced, and peptide barcode proteins are purified from the lysate by immobilization on biotinylated beads. After washing, the beads are treated with TEV protease to release the peptide barcodes, which are filtered and analyzed using LC‐MS/MS to quantify the relative amounts of each peptide and, hence, LNP delivery efficiency.

Peptide barcoding presents various advantages and limitations (Table 2). Unlike FIND, which may overestimate functional delivery by detecting LNP accumulation without confirming mRNA translation, peptide barcoding provides a direct measure of protein expression in target cells. By pairing RNA sequencing with peptide barcoding, both biodistribution and protein expression in target sites (or LNP delivery) can be evaluated in a single experiment, improving predictive accuracy over sequential nucleic acid barcoding and luciferase mRNA validation. Furthermore, peptide barcoding can be applied in any preclinical disease model where the targeted cell type can translate exogenous mRNA into functional protein.

Although peptide barcoding can detect hepatic protein expression at doses as low as 0.001 mg kg^−1^, its sensitivity may be lower than that of DNA or mRNA barcoding due to the lack of a signal amplification step.^[^ ^105^ ^]^ Initial experiments show that quantification of splenic expression of peptide barcodes is less reliable compared to liver lysates, highlighting the need for further improvement of the assay.^[^ ^105^ ^]^ Since peptide barcoding relies on LC‐MS/MS to detect protein expression, it is unsuitable for gene knockdown or silencing applications, where DNA or mRNA barcoding remains preferable.

A key consideration is the potential for non‐linear effects when pooling LNPs with different ionizable lipid species, which may lead to differences in delivery efficiency compared to individual formulations.^[^ ^101^, ^105^ ^]^ Specifically, Rhym et al., found that the ranking of the top three LNPs from peptide barcoding (pooled setting) did not entirely match hEPO assay results (individual setting). However, the top‐ranked LNPs consistently outperformed the original formulation, and the lowest‐ranked LNP produced the least protein.^[^ ^105^ ^]^ Moreover, variation in mRNA translation efficiency across cell types could influence functional delivery predictions‐ an issue inherent to all nucleic acid‐based barcoding strategies.^[^ ^105^, ^120^, ^121^ ^]^ Nonetheless, with broader adoption and improvements, peptide barcoding has the potential to surpass nucleic acid barcoding in evaluating in vivo nucleic acid delivery. Future advancements in proteomic techniques could enhance the sensitivity and precision of peptide barcode quantification, potentially making this approach superior to nucleic acid barcoding for assessing functional delivery.^[^ ^122^ ^]^

## 5 High‐Throughput Characterization Methods

The physicochemical properties of LNPs, including size, surface chemistry, composition, structural integrity, stability, and drug encapsulation efficiency/release kinetics, collectively determine their biological fate and therapeutic efficacy.^[^ ^14^, ^17^, ^123^ ^]^ These properties influence critical pharmacokinetic and pharmacodynamic parameters, such as circulation half‐life, biodistribution, cellular uptake, and endosomal escape efficiency. For example, size and surface chemistry are crucial factors in extending LNP circulation half‐life, minimizing clearance by the mononuclear phagocyte system, and cellular uptake pathways.^[^ ^17^, ^124^ ^]^ The structure of LNPs can impact organ tropism (preferential accumulation in specific tissues).^[^ ^93^, ^125^ ^]^ Additionally, some of these properties are interrelated; for instance, the surface charge of LNPs can influence both stability and aggregation behavior.^[^ ^17^ ^]^ LNP composition, defined by lipid types and molar ratios of constituent lipids, determines membrane integrity and mechanical robustness.^[^ ^12^ ^]^ For ionizable LNPs, the acid dissociation constant (pKa) is arguably the most important property, governing their charge and ionization behavior under physiological and endosomal pH conditions.^[^ ^20^, ^63^ ^]^ This, in turn, affects their in vivo stability, toxicity, biodistribution, delivery, transfection efficiency, and hence therapeutic efficacy.^[^ ^22^, ^126^, ^127^, ^128^ ^]^ LNPs can be designed using ionizable lipids that remain neutral and stable at physiological pH to enhance circulation half‐life by minimizing their interactions with serum proteins and facilitating efficient cellular uptake. Once encapsulated within the acidic environment of endosomes, these ionizable lipids become protonated as the pH drops below their pKa, triggering the destabilization of LNPs and their membrane fusion with the endosome membrane, which releases the therapeutic payload into the cytoplasm.^[^ ^17^, ^19^, ^129^ ^]^ The pKa of LNPs can be tailored by modifying the ratios of ionizable lipid molecules used in their formulation. Optimizing pKa allows precise control over LNP stability during circulation and its triggered destabilization within the acidic endosomal environment, thereby enhancing the cytoplasmic release of therapeutic payloads.^[^ ^128^, ^130^, ^131^ ^]^

Despite the significance of physicochemical properties in determining the therapeutic efficacy of LNPs, conventional low‐throughput techniques remain limited in their ability to screen and optimize formulations from a large LNP library generated from high‐throughput methods. DLS remains a primary method for determining the hydrodynamic size and size distribution of LNPs, with additional capability for measuring their stability across different time points and temperatures. Electrophoretic Light Scattering (ELS) quantifies the surface charge of LNPs in the form of zeta potential–a parameter critical for understanding LNPs' stability and interaction in the physiological environment. Although commercial instruments, such as the Zetasizer Nano‐ZS (Malvern Panalytical), can perform both DLS and ELS in a single system, their sequential sample loading in a single cuvette imposes throughput limitations, restricting the number of formulations that can be analyzed per run. For structural analysis of LNPs, electron microscopy (EM) techniques, particularly cryogenic electron microscopy (cryo‐EM), offer high‐resolution imaging for revealing the internal structure of LNPs.^[^ ^132^ ^]^ However, this method is constrained by extensive sample preparation requirements, low throughput, and time‐intensive data acquisition, making it impractical for rapid iterative formulation screening.^[^ ^14^, ^133^, ^134^, ^135^, ^136^ ^]^

**Figure 8:**
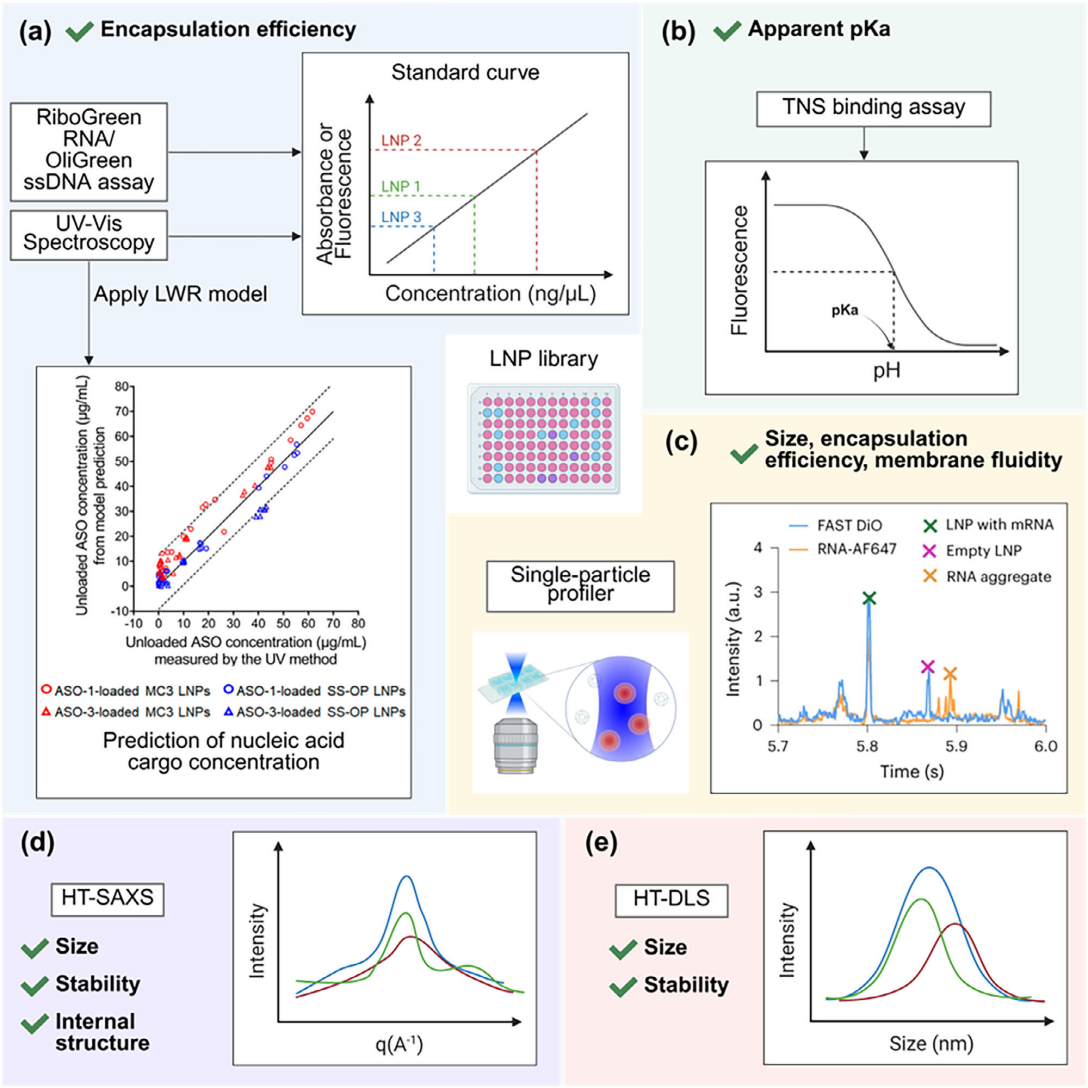
High‐throughput characterization (HTC) methods for LNPs. a) The RiboGreen RNA and OliGreen ssDNA assays enable quantification of EE for RNA and oligonucleotides, respectively. UV–vis spectroscopy can also be combined with model‐fitting approaches to predict cargo EE in situ. LWR = locally weighted regression. Adapted with permission from Ref. [139] Copyright 2022, American Chemical Society. EE is determined by comparing fluorescence or absorbance against a standard curve of intensity vs cargo concentration. b) The apparent pKa of LNPs is determined from the inflection point of fluorescence vs pH plots obtained using the TNS binding assay. c) The single‐particle profiler (SPP) can determine the size, EE, and membrane fluidity at the single particle level by detecting fluorescence fluctuations of particles in each microchannel of a microplate. Adapted with permission from Ref. [140] Copyright 2024, Springer Nature. d) High‐throughput small‐angle X‐ray scattering (HT‐SAXS) yields intensity profiles that reveal structural characteristics of LNPs. e) High‐throughput dynamic light scattering (HT‐DLS) determines the size and size distribution of LNPs in a solution and can be used to monitor LNP stability over time. Created with BioRender.

Recent technological advancements have introduced HTC methods that can keep pace with the large number of LNP libraries generated by microfluidic platforms. For instance, advanced instruments like the CLARIOstar Plus^[^ ^137^ ^]^ and PHERAstar FSX,^[^ ^138^ ^]^ capable of reading microplates with up to 1536 and 3456 wells, respectively, allow rapid measurements across multiple modes such as fluorescence intensity and UV–vis absorbance to determine payload encapsulation efficiency (EE), release kinetics, and pKa of LNPs (**Figure** **8**). These technologies are compatible with various high‐throughput assaying techniques, which can drastically shorten turnaround times and enable researchers to process large sample batches within a short timeframe to identify formulations with optimal physicochemical properties. In this section, our discussion is limited to only HTC methods.

### 5.1 Microplate‐Based Dynamic Light Scattering

Dynamic light scattering is a widely adopted analytical technique for evaluating the size, size distribution, surface chemistry, and stability of LNPs. These physicochemical properties directly influence the pharmacokinetic profiles, biodistribution, and therapeutic efficacy of LNPs. DLS measures the hydrodynamic size of NPs in solution by analyzing the intensity fluctuations of scattered light arising from Brownian motion. This hydrodynamic size accounts for both the core particle size and any attached surface molecules. Tracking size changes over time provides insights into LNP aggregation behavior and stability, both of which are critical for maintaining consistent therapeutic performance. Unlike imaging‐based methods such as cryo‐EM, which require extensive imaging processing and individual particle analysis to measure the average size of LNPs, DLS offers a faster and more streamlined assessment of LNP formulation. Although DLS assumes particles are spherical, and this can introduce minor inaccuracies, this limitation is generally negligible since LNPs are predominantly spherical in structure.

**Figure 9:**
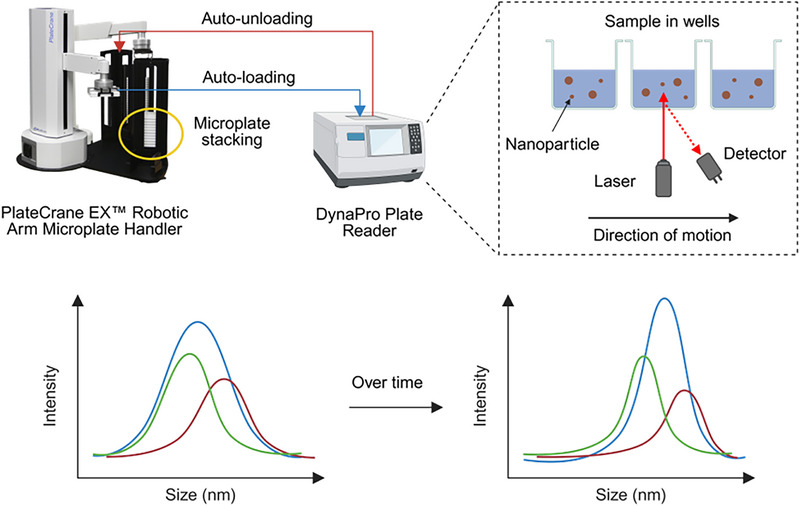
High‐throughput DLS (HT‐DLS) integrated with automated handling. The PlateCrane EX Robotic Arm Microplate Handler allows automated transfer of microplates from a stacked pile into the DynaPro Plate Reader and vice versa. Inside the plate reader, a mobile laser and detector system moves between wells to measure the size and size distribution of NP samples based on the principle of DLS. Image of the Robotic Arm Microplate Handler adapted from Hudson Lab Automation Ref. [143] Created with BioRender.

Conventional DLS, limited by single‐sample cuvette measurements, restricts high‐throughput characterization of LNPs. The multi‐well plate reader (Wyatt Technology, CA, USA) overcomes this limitation by offering high‐throughput DLS technology compatible with microplates of 96, 384, or 1536 wells.^[^ ^36^, ^79^, ^84^, ^94^, ^141^, ^142^ ^]^ This is a technique performed by diluting different formulations of LNPs in PBS to the same concentration and transferring the formulations into individual wells, followed by microplate loading. A mobile laser illuminator and detector measure scattered light intensity from below each well, providing data on hydrodynamic size, size distribution, and stability over time for each sample (**Figure** **9**). This high‐throughput approach enables the collection of large datasets in a single run. This system can be interfaced with robotic liquid handling and automated plate loading systems, eliminating the need for manual intervention (Figure 9).^[^ ^141^, ^142^, ^143^ ^]^ This automation not only improves throughput but also reduces human error and variability, which is essential for consistent data quality. A key advantage of the multi‐well plate reader DLS system is its temperature‐controlled operation, allowing rapid screening of LNP formulations under various storage or physiological conditions. This is particularly useful for investigating LNP stability and aggregation behavior under varying temperature conditions, which is critical for clinical translation. Furthermore, HT‐DLS enables rapid identification of LNP formulations outside of the desirable size range and polydispersity criteria, which are key factors in vivo performance. Oversized or highly polydisperse LNPs are more susceptible to rapid clearance by the mononuclear phagocyte system, limiting circulation time, biodistribution, and therapeutic efficacy. For example, the DynaPro Plate Reader was used to screen 128 LNP formulations in a single run, eliminating 63 candidates that exceeded the target size range (20–200 nm), showed a high polydispersity index (>0.5), or displayed multi‐modal scattering profiles.^[^ ^79^, ^84^, ^94^ ^]^ This allowed the early elimination of suboptimal formulations, thereby streamlining subsequent screening steps and increasing overall experimental efficiency. Beyond size‐based filtering, HT‐DLS has been used to investigate how PEG‐lipid content, total lipid concentration, and nucleic acid loading affect LNP uniformity and stability. In one study, HT‐DLS performed in a fully automated 96‐well format revealed that increasing PEG‐lipid content reduced LNP size but increased polydispersity. An optimal formulation containing 1.5 mol% PEG‐lipid yielded a unimodal size distribution with high encapsulation efficiency.^[^ ^141^ ^]^ Importantly, size and polydispersity index trends identified using HT‐DLS in 96‐well format were strongly correlated (R^2^ >0.9) with data from scale‐up data from microfluidic synthesis, highlighting the predictive power of HT‐DLS in guiding formulation development. Similarly, Sarode et al., also used HT‐DLS to identify optimal formulation for nucleic acid delivery from a library of 54 antisense oligonucleotides (ASOs) loaded LNPs with varying PEG‐lipid composition.^[^ ^144^ ^]^ The resulting dataset revealed clear correlations between PEG content and LNP diameter, and that more monodisperse LNPs were observed at higher PEG molar ratios. The results also showed that LNPs sized between 100 and 150 nm exhibited the highest mRNA knockdown in primary neurons. These finding links HT‐DLS‐based size selection to functional delivery outcomes. Collectively, these studies demonstrate that HT‐DLS serves as a powerful pre‐screening filter, enabling early elimination of suboptimal candidates before functional evaluation, and thereby accelerating the rational LNP design.

Nevertheless, the multi‐well plate reader‐based DLS technology lacks integrated electrophoretic light scattering (ELS) capabilities, limiting simultaneous measurement of surface charge–an important parameter of colloidal stability in a biological environment. Future technological advancements should focus on integrating DLS with ELS within a single high‐throughput analytical platform that enables concurrent characterization of size, stability, and surface charge.

### 5.2 High‐Throughput Small‐Angle X‐Ray Scattering

**Figure 10:**
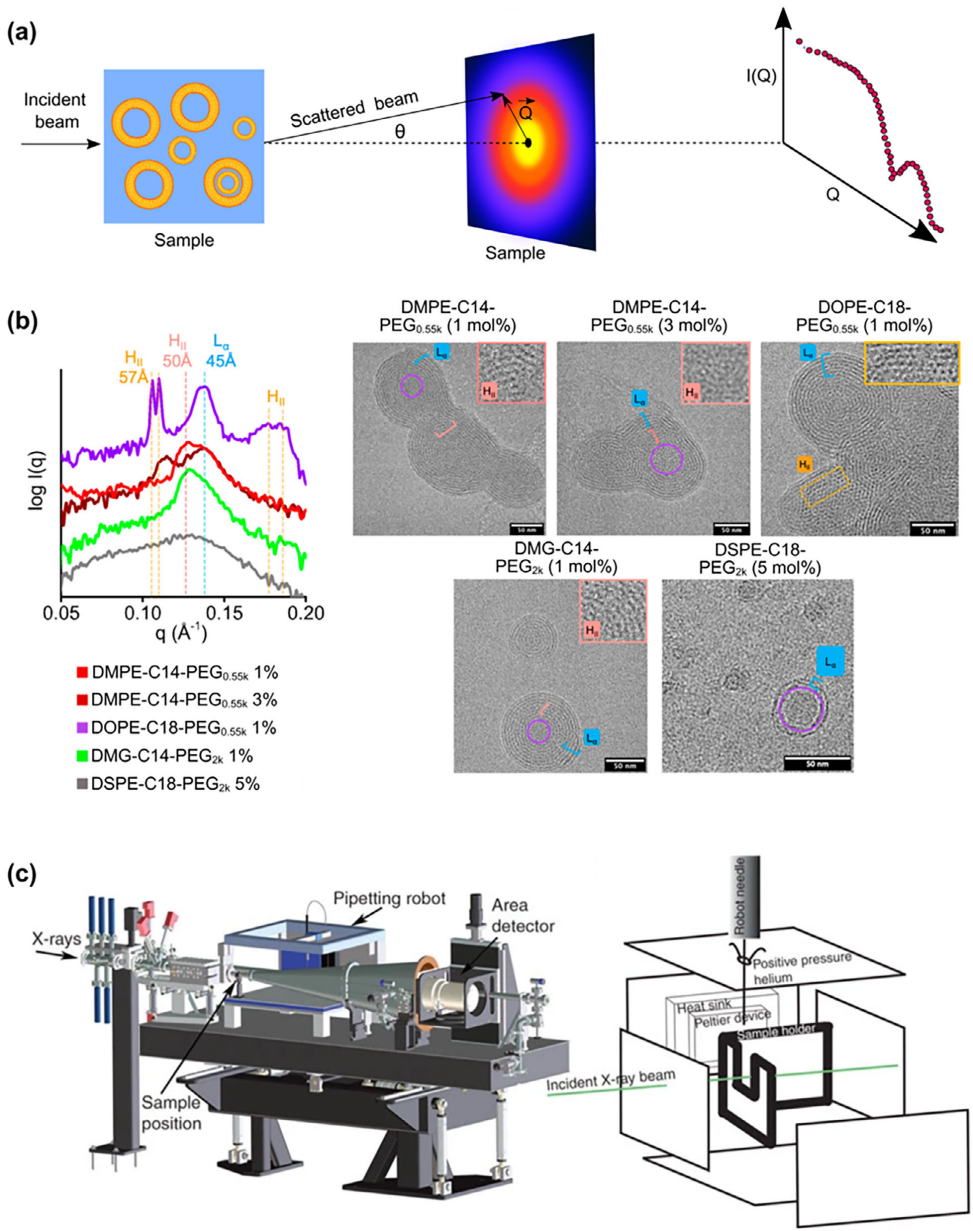
High‐throughput small‐angle X‐ray scattering (HT‐SAXS) for structural analysis of LNPs. a) Schematic of a small‐angle scattering set‐up. X‐rays scattered by lipid vesicles produce an interference pattern on a 2D detector, which is radially averaged to generate 1D intensity (I(Q)) vs scattering vector (Q). Reproduced with permission from Ref. [154] Copyright 2024, Elsevier. b) Representative SAXS profiles (left) of five LNP formulations synthesized using different PEG‐lipid, showing characteristic diffraction features linked to internal structures. Diffraction peaks corresponding to internal structural phase: hexagonal (H_II_) at d≈57 A° (orange dashed lines) and 50 A° (pink dashed lines), and lamellar (L_α_) phases at d≈45 A° (blue dashed line). The sharpness and location of the diffraction peaks indicate the degree of internal structure order. For example, the formulation containing DOPE‐C18‐PEG_0.55k_ (purple) displays sharp and well‐defined H_II_ peaks, indicating a highly ordered internal structure, while DSPE‐C18‐PEG_2k_ (gray) shows a broad scattering peak, which is characteristic of a disordered structure. Corresponding cryo‐EM images (right) of the same LNPs reveal distinct compartmentalization of the ASOs‐lipid within H_II_ (red), crystalline H_II_ (yellow box), L_α_ (blue scale), and disordered phases (violet circle). A magnified view (insets in Cryo‐EM images) highlights ASO‐lipid compartmentalization in the H_II_ phases of some LNPs. Adapted with permission from Ref. [14] Copyright 2023, American Chemical Society. c) Schematic of an HT‐SAXS setup. Samples from a microplate are automatically loaded by a robotic liquid handler into a temperature‐controlled cell. An incident X‐ray beam passes through each sample, and the scattered X‐rays are collected on a 2D detector. Helium purging minimizes air scattering and prevents oxidative damage to the samples, ensuring high‐quality data acquisition in a high‐throughput format. Reproduced with permission from Ref. [158] Copyright 2009, Springer Nature.

Small‐angle scattering (SAXS) has become an essential analytical method for elucidating the internal structure of LNPs, providing insights into their nanoscale organization, including phase behavior, the degree of crystallinity, and core–shell architectures.^[^ ^14^, ^133^, ^145^, ^146^, ^147^, ^148^, ^149^, ^150^, ^151^ ^]^ By probing variations in electron density within the LNP, SAXS generates scattering profiles that indicate the presence of ordered domains (e.g., lamellar or inverse hexagonal phase) through distinct diffraction peaks, while disordered or amorphous regions appear as broader and less defined scattering features. In addition to internal structure, SAXS can also evaluate the size and stability of LNPs under varying environmental conditions, as well as the loading and the spatial distribution of therapeutic agents.^[^ ^152^ ^]^ In a typical SAXS experiment, exposing a sample to X‐rays that are scattered to different degrees due to electron density variations within the LNPs generates scattering profiles with distinct peaks and shoulders.^[^ ^153^, ^154^ ^]^ The resulting scattering intensity (I) profile is plotted against the scattering wavevector (q), where *q = (4π sin θ)/λ*, with 2θ representing the scattering angle and λ the wavelength of the X‐rays (**Figure** **10**a).^[^ ^14^ ^]^ The inverse relationship between q and the characteristic length of the scattering object (d), given by *q = 2π/d*, enables SAXS to resolve nanoscale structural features such as interlayer spacing, lipid ordering, and core–shell architecture.^[^ ^148^ ^]^ Advanced model fitting of scattering profiles further allows the quantification of parameters such as shell thickness, internal lattice spacing, and radial density distributions, thereby linking structural features to LNP stability and functionality.^[^ ^155^, ^156^, ^157^ ^]^ When complemented by real‐space imaging methods such as cryo‐EM, SAXS profiles can be validated and used to build robust structural models (Figure 10b).^[^ ^135^ ^]^

Historically, SAXS was a relatively low‐throughput technique. Each sample had to be loaded and measured individually, often at specialized synchrotron beamlines or using lab‐scale instruments with lengthy exposure times. Recent advances in high‐throughput SAXS (HT‐SAXS), such as the SIBYLS beamline 12.3.1 at the Lawrence Berkeley National Laboratory, have accelerated the structural analysis of LNPs.^[^ ^158^, ^159^ ^]^ Unlike conventional SAXS, which processes single samples per execution,^[^ ^14^, ^133^, ^149^, ^160^ ^]^ HT‐SAXS can analyze 96 samples in less than 4 h using microplate‐based sample handling (<30 µL per well).^[^ ^158^, ^160^, ^161^ ^]^ This platform incorporates a liquid‐handling robot that automates sample loading, exposure, retrieval, and cleaning cycles; significantly reducing both data acquisition time and human error compared to conventional SAXS workflows (Figure 10c).^[^ ^158^, ^160^ ^]^ HT‐SAXS capabilities are available at several synchrotron facilities worldwide. The Australian Synchrotron houses the BioSAXS beamline, equipped with a liquid handling system (Coflow Autoloader) that enables high‐throughput analysis across a broad particle size range (≈0.001–3 Å^−1^) while maintaining low background noise.^[^ ^162^ ^]^ Another HT‐SAXS facility is the Synchrotron P12 beamline of the European Molecular Biology Laboratory (EMBL) located at the PETRA III storage ring (DESY, Hamburg),^[^ ^163^, ^164^, ^165^ ^]^ which can collect scattering data with exposure times as short as 30–50 ms, spanning structural features from 0.5 to 300 nm. It also employs robotic sample changers and an integrated pipeline (SASFLOW) for intermediate data processing, delivering Guinier analyses and low‐resolution models within minutes. The facility allows researchers to send samples for analysis and interpretation.^[^ ^161^, ^166^ ^]^

The significance of HT‐SAXS in LNP screening is demonstrated by a recent study directly linking LNP internal structure to functional outcomes. Hammel et al., used HT‐SAXS (SIBYLS beamline 12.3.1) to demonstrate a direct correlation between the internal structure of antisense oligonucleotides (ASOs)‐loaded LNPs and their gene silencing activity.^[^ ^14^ ^]^ By screening a large library of 54 LNP formulations (varying in PEG‐lipid content) using HT‐SAXS, they revealed important structural differences in ASOs‐loaded LNP formulations, notably the presence of hexagonal (H_II_) and lamellar (L_α_) phases, as well as distinction between ordered (crystalline) and disordered or fluid‐like behavior (Figure 10b, left). These structural characteristics, initially observed in SAXS profiles, were cross‐validated using cryo‐EM imaging of selected formulations (Figure 10b, right), confirming the presence of hexagonal lattices or multilamellar structures as indicated by the scattering patterns. Importantly, the study showed that the ratio of disordered to ordered lipid phase, as quantified from the SAXS profiles, was strongly predictive of gene silencing activity. Formulations with lower disordered/ordered ratios (i.e., more highly ordered ones) achieved greater mRNA knockdown, whereas those with predominantly disordered interiors were less effective. By screening many formulations, the study identified that certain PEG‐lipid compositions produced a highly ordered internal structure, which corresponded to the best‐performing formulations in biological assays. This highlighted HT‐SAXS as not just a structural screening tool, but a powerful method to predict and optimize LNP biological performance based on the internal architecture (structure‐function relationship). In practical terms, this can reduce the burden of in vivo testing. Instead of injecting dozens of candidates to see which works best, one can first use HT‐SAXS (alongside in vitro assays) to narrow the field to a few promising LNPs with optimal internal architecture for efficacy.

Despite its advantage, challenges remain in SAXS data interpretation, particularly in cases where broad NP size distributions and the coexistence of multiple internal phases can introduce signal variability,^[^ ^14^, ^154^ ^]^ complicating the interpretation of subtle structural differences. Moreover, SAXS peak assignments may vary between studies due to differences in model assumptions and sample conditions.^[^ ^149^, ^150^ ^]^ To address these issues, it is important to employ model‐based fitting strategies and complement SAXS with orthogonal techniques such as cryo‐EM or DLS. Cryo‐EM provides direct visualization of NP morphology and internal structure, confirming phase identities obtained from SAXS, while DLS serves as a rapid pre‐screening tool to assess NP size and aggregation status before SAXS measurement. Machine learning models trained on simulated and experimental SAXS data can enhance data reliability and accuracy.

### 5.3 Microplate‐Based Spectroscopic Techniques

Microplate‐based spectroscopic methods, particularly UV–vis and fluorescent assays, have emerged as indispensable tools for rapid and high‐throughput characterization of LNPs. These techniques greatly enhance the efficiency of iterative LNP formulation optimization. They allow rapid assessment of several samples, facilitated by automated liquid handling and multi‐well microplate readers (ranging from 96 to 1536 wells) and automated liquid handling systems to facilitate the rapid processing of large sample sets. These tools provide insights into the physicochemical characteristics of LNPs, such as encapsulation efficiency, cargo release kinetics, stability (or aggregation state), surface chemistry, and size and size distribution.^[^ ^141^ ^]^ UV–vis spectrophotometry operates on the principle of molecular absorption at specific wavelengths, which is unique to different therapeutic molecules. For instance, nucleic acids exhibit a peak absorbance of ≈260 nm, enabling their quantification with high sensitivity.^[^ ^36^, ^141^ ^]^ This method is particularly useful for determining encapsulation efficiency (EE), which is the ratio of encapsulated material to total (free and encapsulated) material, given by the formula *EE = (Amount of encapsulated material/Total amount of material) × 100%*. In practice, unencapsulated molecules are separated from LNPs (e.g., dialysis or centrifugation), and UV–vis absorbance is measured to determine EE. The method is easily scalable for high‐throughput characterization, enabling the analysis of up to 1536 samples per plate to guide the iterative refinement of LNP formulation with large EE.

Advances in multi‐mode microplate readers have further expanded the capabilities of high‐throughput LNP characterization, allowing detection across multiple spectroscopic modalities, including absorbance, fluorescence, time‐resolved fluorescence, and luminescence. Fluorescence‐based detection in multimode readers is also widely employed to quantify encapsulation efficiency and release of payloads in LNPs using different fluorescent dyes (Figure 8).^[^ ^167^, ^168^ ^]^ The fluorescence intensity of hundreds of samples can be measured in a single run.^[^ ^167^ ^]^ The RiboGreen RNA assay has been widely used to determine RNA encapsulation efficiency in LNP formulations, using selective fluorescent signal enhancement of RiboGreen upon binding to nucleic acid.^[^ ^36^, ^52^, ^85^, ^134^, ^150^ ^]^ In this assay, free RNA is measured by directly adding RiboGreen solution to buffer‐diluted LNPs, and total RNA is determined by adding Triton X‐100 solution, which destabilizes LNPs to release encapsulated RNA.^[^ ^168^ ^]^ Alternatively, LNP samples can first be dialyzed to isolate free RNA before the assay, then treated with Triton X‐100 solution to release the encapsulated RNA.^[^ ^150^ ^]^ The total amount of RNA can be obtained by summing the free and encapsulated material. The quantity of nucleic acids is then measured using standard calibration curves with known concentrations of nucleic acid solutions.^[^ ^52^, ^150^ ^]^ Furthermore, the encapsulation efficiency is calculated as a percentage of encapsulated to total nucleic acids. A similar approach has been applied to quantify oligonucleotide encapsulation using the OliGreen ssDNA assay.^[^ ^14^, ^139^, ^169^ ^]^ These fluorescence‐based assays offer high sensitivity (ng/mL detection limits) and compatibility with high‐throughput processes, making them an efficient method for iterative formulation refinement.^[^ ^170^, ^171^ ^]^

Recent advancements have led to the introduction of the single‐particle profiler (SPP), a high‐throughput analytical platform developed for the characterization of LNPs at the single NP level, ranging from 5 to 200 nm.^[^ ^140^ ^]^ Unlike conventional methods that provide population‐averaged data, SPP uses fluorescence fluctuation intensity analysis, whereby individual fluorescently labeled LNPs diffuse through a confocal volume, allowing measurement of fluorescence intensity fluctuation and diffusion coefficients. When combined with a 96‐well microplate and an automated stage movement imaging program, the SPP technique facilitates simultaneous analysis of thousands of individual LNPs per well, allowing systematic multiparametric screening of size, encapsulation efficiency, and membrane fluidity. These properties are measured across two profiling modes, each requiring 15 s per technical replicate, with 40 replicates per well or sample, ultimately resulting in a total measurement time of ≈20 min per well/sample. Size estimates are derived by comparing DLS size measurements with diffusion coefficients measured by the SPP and fitting to the Stokes–Einstein equation. The number of full and empty LNPs is determined by measuring the co‐occurrence of mRNA and dyed‐lipid signals for each particle, while membrane fluidity is assessed by analyzing spectral fluctuations of the lipid‐dye. This technique with single NP level resolution significantly improves the precision and depth of characterization datasets, streamlining formulation optimization. Despite demonstrating high reliability in measurements such as LNP size (which fits the Stokes–Einstein equation) and mRNA encapsulation (with co‐occurrence approaching 100%), further cross‐validation with other characterization tools should be performed to confirm the technical capabilities of the SPP.

It should be noted that conventional quantification methods are limited in their applicability for high‐throughput quantification since they require the use of complex and time‐consuming sample preparation, such as separating unloaded molecules from cargo‐loaded LNPs. However, recent advances in machine learning‐driven spectroscopic data analysis have enabled nucleic acid quantification without separation steps. For example, Fan et al., introduced a locally weighted regression model to directly analyze full UV–vis spectral data from microplate readers, enabling accurate nucleic acid quantification without prior payload separation.^[^ ^139^ ^]^ This strategy not only simplifies the analysis but also reduces sample handling errors and processing time, improving overall analytical reproducibility.

In addition to payload quantification, microplate‐based UV–vis spectroscopic measurements can assess LNP stability and aggregation states by monitoring optical density (OD) changes at wavelengths between 400 and 600 nm. A stable dispersion maintains constant OD values, whereas aggregation or fusion events induced by salts (e.g., Ca^2+^ and Mg^2+^) are readily detected by increased OD.^[^ ^172^, ^173^ ^]^ Furthermore, it has been shown that the size of LNPs may also be evaluated by light scattering, with smaller LNPs displaying high absorption at lower wavelengths and larger LNPs scattering more light, shifting the absorbance peak to a higher wavelength.^[^ ^174^ ^]^

**Figure 11:**
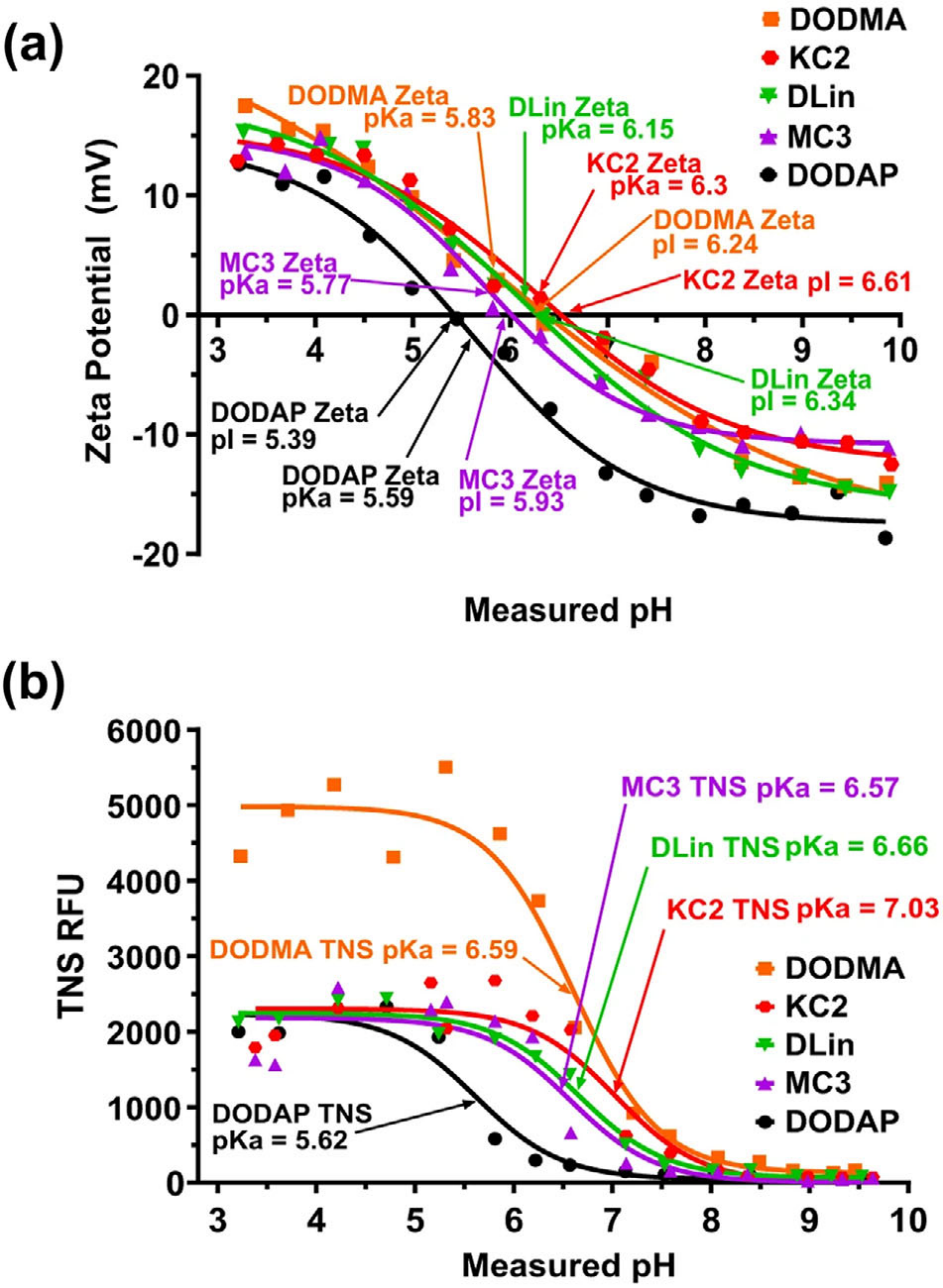
Determination of pKa values of mRNA LNPs formulated with different commercially available ionizable lipids. a) Zeta potential measurements of LNPs as a function of pH (3–10) revealed the transition from positively to negatively charged NP. The isoelectric point (pI) is defined as the pH at which the net surface charge is zero. b) Apparent pKa values of LNPs determined using the TNS binding assay, which detects the onset of lipid protonation above pH≈6. Data were fitted using the Henderson‐Hasselbalch model. Reproduced from Ref. [20] Copyright 2021, Springer Nature.

Another critical parameter, the apparent acid dissociation constant (pKa) of ionizable LNPs, influences LNP surface charge, stability, cellular uptake, and endosomal escape efficiency.^[^ ^128^ ^]^ The term “apparent pKa” is used since the measured pKa value of LNPs differs from the intrinsic pKa of the same lipids in the solution. This shift occurs due to the complex microenvironment within LNPs, where factors such as lipid packing, surface charge, and interaction with surrounding lipids or encapsulated cargo can influence protonation behavior. To achieve the maximum therapeutic index, the apparent pKa value of LNPs should be optimized. The apparent pKa of LNPs can be measured in a high‐throughput manner using the 2‐(p‐toluidino)‐6‐naphthalenesulfonic acid (TNS) binding assay.^[^ ^36^, ^64^, ^84^, ^134^ ^]^ In the free aqueous solution, the TNS molecule is non‐fluorescent; upon coming into contact with a cationic lipid species, it fluoresces.^[^ ^126^, ^175^ ^]^ As TNS molecules potentially bind to the surface of LNPs without significant penetration into the inner compartments, values derived from this assay likely indicate the pKa properties of accessible ionizable lipids at the LNP surface, which is the apparent pKa.^[^ ^22^ ^]^ LNPs have been tested in buffer solutions with varied pH. After mixing buffer solutions, LNPs, and TNS solution in 96‐ or 384‐well plates and incubating under gentle shaking, the fluorescent intensity was measured with a microplate reader at excitation wavelengths of ≈325 nm and emission wavelengths of 435 nm.^[^ ^84^, ^128^ ^]^ By locating the inflection point on the generated fluorescence against pH curve,^[^ ^128^, ^176^ ^]^ the apparent pKa of LNPs can be determined. Alternatively, the zeta potential of LNPs measured at different pH levels can be used to determine the pKa at the inflection point (**Figure** **11**). Nevertheless, the low data acquisition rate of electrophoretic light scattering (ELS) makes high‐throughput zeta potential measurement difficult. The TNS assays, in contrast, have been adapted for high‐throughput formats through the integration of acoustic liquid handling systems. For instance, Cui et al., demonstrated a streamlined workflow using a 384‐well plate format and the ECHO 550 system to automate LNP pipetting, pH titration, and TNS addition, significantly improving assay throughput.^[^ ^36^ ^]^ Furthermore, previous studies have shown that TNS‐derived pKa values align more closely with the theoretically predicted pKa values than those obtained from zeta potential measurement.^[^ ^20^ ^]^ Given its higher sensitivity, accuracy, and adaptability to automation, the TNS assay is the most reliable approach for determining the pKa of ionizable LNPs.^[^ ^20^, ^175^ ^]^

## 6 Lipid Nanoparticle Development Pipeline

**Figure 12:**
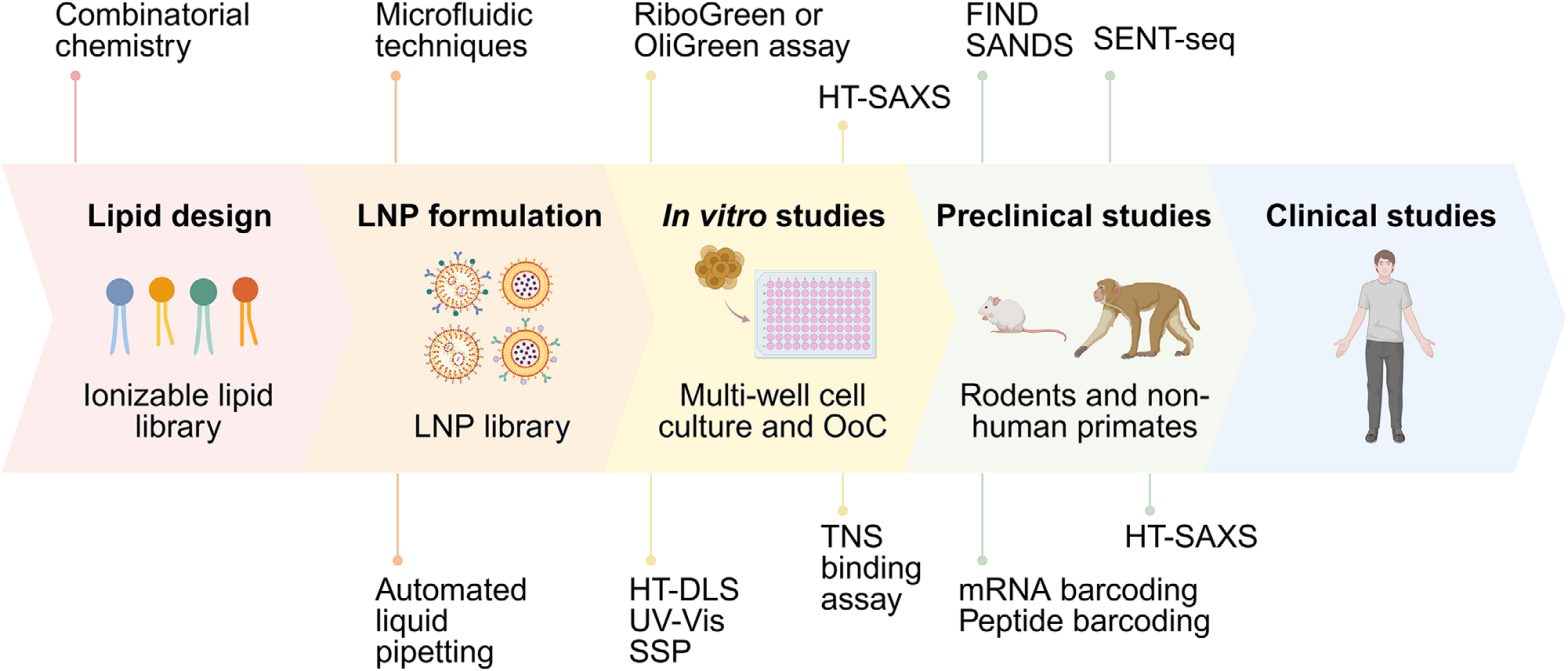
Lipid nanoparticle development pipeline. In the formulation stages, ionizable lipids are synthesized via combinatorial chemistry, which are then formulated into LNPs using microfluidics and automated liquid pipetting methods. Before in vitro studies, HTC techniques such as DLS, UV–vis spectroscopy, RiboGreen, and OliGreen assays. and SPP are applied to screen out suboptimal formulations. In vitro and in vivo HTS techniques enable biodistribution and transfection efficiency studies. Key physicochemical parameters that influence LNP performance can be further characterized using HT‐SAXS and TNS binding assays.

High‐throughput combinatorial synthesis, together with high‐throughput screening and analytical characterization, shows great promise in transforming the development of LNP‐based delivery systems by streamlining the process of filtering large formulation libraries (**Table** **3** and **Figure** **12**). In a standard LNP development pipeline, as shown in Figure 12, a large combinatorial library of candidate ionizable lipids is first generated using high‐throughput synthetic chemistry. Various reactions (e.g., Michael addition and multicomponent Ugi reactions) can generate several tens of chemically and structurally diverse ionizable lipids within a few days.^[^ ^52^ ^]^ These lipid libraries are then formulated into LNP libraries by high‐throughput microfluidic techniques. Microfluidic mixers offer highly controlled, reproducible assembly of LNPs and can be parallelized to produce numerous formulations under identical conditions. Beyond varying the ionizable lipid, LNP libraries can be diversified by tuning other formulation parameters, such as PEG‐lipid type and ratio. To further enhance the synthetic throughput, robotic liquid handlers can be employed to automate formulation procedures, reducing manual labor. This approach significantly increases the efficiency of lipid and LNP production from tens of formulations in days to over a thousand per day. Together, combinatorial lipid synthesis and parallel LNP formulation establish a large initial library for screening.

**Table 3:** Summary of high‐throughput synthesis, screening, and characterization techniques. A comparative overview of HTC and HTS strategies for LNPs, illustrating their technological capabilities and advantages/limitations.

Technique	Key Parameters Measured	Throughput Potential	Automation Compatibility	Accuracy	Advantages	Limitations
	In Vitro High‐Throughput Screening					
Mult‐well cell culture	Cell cytotoxicity, mRNA transfection efficiency, etc.	Up to 1536 formulations per experiment	Yes	Varies depending on the culture model complexity	Simple and cost‐efficient for quick combinatorial library screening	Costly to scale up model complexity; lack of standard in vitro models
NanoPRISM	Cellular association, nanoparticle uptake	Up to 1536 formulations per experiment	Yes	Varies depending on culture model complexity and cell‐type‐dependent barcoding efficiency	Assesses LNP cellular association across hundreds of cell types	Cannot measure functional delivery; measurement accuracy may be inconsistent across cell types
	In Vivo High‐Throughput Screening					
DNA barcoding	Nanoparticle biodistribution	Hundreds to thousands per animal	No	Biodistribution: High Functional delivery: Low	High sensitivity; suitable for all kinds of gene‐based therapy	Poor predictor of functional delivery; LNP properties may be affected by cargo type
mRNA barcoding	Nanoparticle biodistribution; mRNA functional delivery	Hundreds to thousands per animal	No	Biodistribution: High Functional delivery: Moderate	High sensitivity; structurally similar to native mRNA	Unsuitable for gene silencing or knockdown applications
Peptide barcoding	Nanoparticle biodistribution; mRNA functional delivery	Hundreds to thousands per animal	No	Functional delivery: High	Predicts functional delivery	Requires additional RNA sequencing for biodistribution studies; lower sensitivity than other barcoding methods
	High‐Throughput Characterization					
HT‐DLS (± Automated microplate handler)	Size, size distribution, stability	Up to 1536 formulations per run	Yes	High	Suitable for automation	No zeta potential measurements; assumes spherical shape; polydispersity inflation with serum
HT‐SAXS (In‐built automated liquid handler)	Size, stability, internal structure, phase behavior, lattice spacing, drug loading	96 samples in ≈4 h	Yes	High but requires cross‐validation with cryo‐EM	Structural insights, semi‐automation enabled by a liquid handler	Complex data interpretation requires high‐end technology.
UV–vis Spectroscopy (± Model fitting approach)	EE, stability, aggregation	Up to 3456 formulations per run	Yes (with appropriate automated liquid handler)	Moderate	Simple, scalable, computer‐assisted prediction is possible	Interference from overlapping absorbance peaks; lower detection limits than fluorescent binding assays
Fluorescent Assays (e.g., RibroGreen and OliGreen)	EE, release kinetics	Up to 3456 formulations per run	Yes (with appropriate automated liquid handler)	High for overall encapsulation	High sensitivity; compatible with automation	Requires dye‐labeled or nucleic acid cargo; averaged data cannot reveal empty LNPs
TNS Binding Assay (+ automated liquid handler)	Apparent pKa	Up to 384‐1536 formulations per run	Semi	Limited, sensitive to preparation conditions	Reliable; potential for semi‐ to full automation	Measures only the surface pKa, may miss bulk behavior
SPP (+ 96‐well microplate + automated stage movement)	Size, EE, membrane fluidity	≈20 min per sample	Yes	High (≈100 %) for encapsulation High for size measurements	Single‐particle resolution; multiparametric	Requires fluorescence; complex instrumentation

High‐throughput screening and characterization methods are then employed to systematically narrow down the LNP library through successive stages of in vitro and in vivo evaluation. LNP libraries must then be screened for their biodistribution and transfection efficiencies. Preliminary physicochemical screens filter out suboptimal LNP candidates that display undesirable physicochemical properties and high sample heterogeneity. HT‐DLS can automatically size and assess the polydispersity of tens to hundreds of formulations, quickly eliminating those with undesirable size (e.g., overly large or polydisperse particles) or poor colloidal stability. Fluorescent nucleic‐acid binding assays (such as RiboGreen or OliGreen for RNA/DNA) and UV–vis measurements are used to quantify encapsulation efficiency, flagging formulations that fail to encapsulate sufficient cargo. A recently developed single‐particle profiler (SPP) method can further characterize nanoparticle populations by measuring individual LNP properties (e.g., per‐particle mRNA content and size). While this single‐particle analysis shows great potential for multiparametric characterization of LNP heterogeneity, it remains an emerging technique requiring additional validation before widespread adoption. These HTC techniques provide preliminary insights about LNP properties, narrow down the number of formulations for downstream studies, and conserve resources for studying high‐performing formulations. At this early stage, these HTC tools yield rapid go/no‐go criteria, for example, eliminating unstable or poorly loaded LNPs, thereby focusing resources on a smaller subset of promising formulations for biological screening. In vitro HTS methods serve as efficient preliminary tools to screen combinatorial lipid libraries and identify high‐performing formulations for targeting specific cells or tissues, further reducing the size of LNP libraries for in vivo screening. Standard multi‐well plate cell culture and NanoPRISM predict the transfection efficiency and selectivity of LNP cell/tissue accumulation with debatable accuracy due to the oversimplicity of 2D culture models. Fortunately, the predictive accuracy of in vitro HTS strategies can be improved through advanced biomimetic cell culture models (e.g., humanized organ‐on‐a‐chip), which better recapitulate physiological conditions.

At the preclinical level, in vivo HTS methods using barcoding technologies have demonstrated remarkable potential in elucidating biodistribution on a large scale using small‐scale animal models. By allowing simultaneous evaluation of hundreds of formulations within a single animal model, these strategies overcome the inherent limitations of conventional low‐throughput in vivo studies and provide a robust framework for optimizing LNP formulations before clinical validation. DNA barcoding strategies like FIND simultaneously study biodistribution and transfection efficiency in transgenic mouse models, while SANDS improves on this by eliminating the need for transgenic mouse models. Since regulatory guidelines recommend that biodistribution studies be conducted across various species,^[^ ^177^ ^]^ typically from mice to non‐human primates (NHPs),^[^ ^107^, ^108^ ^]^ SENT‐seq, having been used in NHPs, offers an alternative for single‐cell analysis of LNP performance in larger species. mRNA and peptide barcoding strategies may further enhance the accuracy of assessing mRNA functional delivery compared to DNA barcoding strategies, but have yet to see widespread adoption, and their feasibility in NHPs remains untested.

HTC methods are essential for understanding structure‐function relationships in LNP development, especially after identifying hits from in vitro and in vivo screening. Preliminary HTC data obtained during initial screens can be mapped onto functional hits to determine application‐specific optimal sizes. For deeper physicochemical optimization of LNP formulations, HT‐SAXS enables rapid structural profiling of dozens of formulations within hours, facilitating the identification of key features that correlate with high transfection efficiency. The TNS binding assay, used to assess apparent pKa values of LNPs, supports ionizable lipid optimization by linking pKa to functional delivery performance. While it can be performed pre‐screening to eliminate formulations with pKa values significantly out of the effective range of pH 6–7, it is potentially more efficient when applied post‐screening to focus only on top‐performing candidates. In summary, incorporating high‐throughput methods at each stage of LNP development from combinatorial synthesis (to explore chemical diversity) to parallel formulation (to generate LNP libraries), through in vitro and in vivo HTS (to efficiently evaluate function in relevant models), and finally HT characterization (to rationalize and refine lead formulation properties), can dramatically accelerate the development of LNP delivery systems.

## 7 Future Perspectives

As we move forward, high‐throughput techniques will continue to uncover key scientific insights (structure‐function relationships and therapeutic performance) and serve as a critical platform for accelerating the translation of LNP nanomedicine from bench to bedside. To achieve this, several key avenues should be taken into consideration. First, as HTS and HTC methods become increasingly significant to LNP research, scaling up these techniques and making them accessible to the broader scientific community will be imperative. Although recent progress has enabled the screening of LNP formulations on the order of 100 LNPs, future efforts are required to expand this capacity to tens of thousands of formulations. Achieving this will require further miniaturization (i.e., reducing sample volume through microfluidic synthesis and high‐density multi‐well plate readers) and parallelization (i.e., simultaneous multi‐sample analysis and barcoding strategies) to enhance efficiency and scalability. Automation will continue to advance HTS and HTC methodologies for streamlining LNP formulations, with particular emphasis on integrating formulation, characterization, and/or screening and analysis. Future HTS and HTC platforms are likely to be integrated with microfluidic devices to synthesize LNP libraries that are immediately subjected to in‐line HTC characterization and biological assays (either in cells or tissues), with minimal human intervention. This integration will drastically reduce iteration times, as thousands of formulations can be made and tested in a day. Such high‐throughput formulation and characterization tools dramatically accelerate design‐of‐experiment cycles, while using an order of magnitude less material than traditional methods.

Second, there is a need to scale up the complexity of in vitro HTS models, e.g., incorporating 3D cell culture, organoids, or even ex vivo organs in a multi‐well format to better mimic in vivo biology. A major challenge in LNP research remains the high failure rate of formulations in clinical trials, due to issues surrounding the reliability of data obtained from murine models. While regulatory frameworks encourage the use of NHPs in pre‐clinical studies, they are limited by cost, ethical concerns, and practical demands.^[^ ^177^, ^178^ ^]^ Additionally, LNPs may lose efficacy when scaled up in NHPs, necessitating LNP reformulation to enhance therapeutic performance.^[^ ^108^ ^]^ Advanced in vitro models with high predictive accuracy can therefore relieve the burden of conducting cost‐ and labor‐intensive in vivo studies. This can be achieved by converging microfluidic LNP production with physiologically relevant screening models, for instance, organ‐on‐a‐chip models of human tissues. Automated platforms could produce LNP libraries and perfuse them through microfluidic tissue constructs (such as a brain‐on‐a‐chip or tumor‐on‐a‐chip), allowing the seamless, downstream evaluation of which formulation penetrates and delivers the therapeutic payload most efficiently. Although progress is being made, challenges remain in adapting these complex systems to high‐density plate formats while ensuring compatibility with automated liquid handling technologies. Moreover, overcoming problems related to reproducibility and data variability when using complex biological models at scale should be prioritized to enhance the reliability of screening outcomes.

Third, integrating scRNA‐seq and ST with in vivo barcoding strategies can provide more information about LNP biodistribution and functional delivery data. scRNA‐seq profiles thousands of individual cells in treated tissue to identify cell types and any delivered transcripts/barcodes, while ST captures local gene expression data to reveal the cell types present within tissue niches. The work by Lehrich et al., revealed transcriptional reprogramming in tumor hepatocyte subtypes and upregulation of interferon in immune cells.^[^ ^117^ ^]^ ST confirmed the loss of β‐catenin‐rich, emergence of reprogrammed hepatocyte subtypes, as well as localized immune infiltration in tumor areas, aligning with scRNA‐seq data. This example demonstrates the power of cross‐validating both datasets for analyzing LNP spatial distribution. Barcoding strategies, though a powerful technique for evaluating LNP biodistribution, often rely on cell‐sorting methods (e.g., FACS) to isolate successfully transfected cells from processed tissue, resulting in a loss of spatial information. A combined workflow could involve administering barcoded LNP pools to an animal, performing scRNA‐seq for high‐resolution cell‐type identification and barcode recovery, and then applying ST in a parallel specimen to reveal where those transfected cell populations reside. Computational integration tools then align the cell types and barcode information to the spatial map by comparing the expression levels of common genes and co‐expression patterns across the scRNA‐seq and ST data, thus revealing the precise location of LNP functional delivery. Such integrated strategies extend beyond descriptive biodistribution, providing mechanistic insight that can inform iterative LNP design.

Fourth, AI and ML models are emerging as powerful tools in LNP design, though their role is still evolving from retrospective analysis toward true predictive optimization. Recent advances in ML models have demonstrated their ability to accelerate LNP discovery.^[^ ^61^, ^179^, ^180^, ^181^ ^]^ For example, random forest regression models have been used to predict mRNA delivery by identifying phenolic headgroups and lipid parameters (constituent ratio and tail length) on delivery efficiency, enabling data‐driven LNP design.^[^ ^182^ ^]^ Current ML models can analyze experimental data to identify key features of successful LNPs. However, these current ML models for LNPs remain largely descriptive rather than fully predictive. For instance, models often investigate experimental data to find correlations, but translating those correlations into reliable predictions for complex biological environments is nontrivial. A fundamental limitation is the scarcity of large and high‐quality datasets for the LNP‐based delivery platform. Therefore, larger and more diverse training datasets should be established by pooling HTC and HTS datasets from multiple studies, allowing ML algorithms to identify structure‐function relationships.

**Figure 13:**
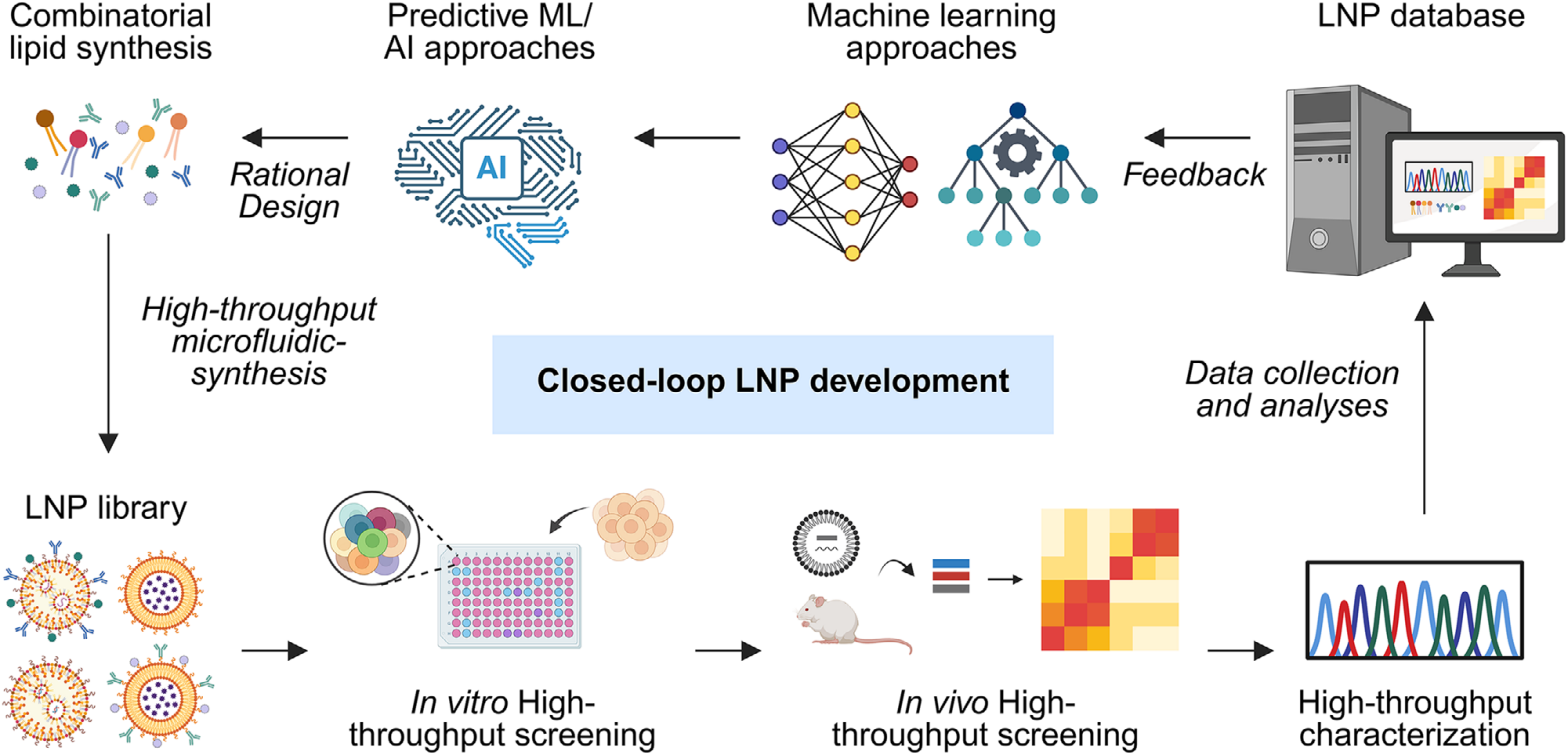
Closed‐loop pipeline integrating high‐throughput strategies and machine learning for optimizing LNP formulation. Combinatorial formulation strategies coupled with high‐throughput microfluidic synthesis allow the rapid generation of large LNP libraries. These formulations are subjected to HTC and in vitro/in vivo HTS strategies to assess their properties, such as size, structure, size distribution, stability, cellular uptake, intracellular delivery, biodistribution, and therapeutic efficacy. Experimental data are collected and analyzed to establish structure‐function relationships and populate the LNP database. ML algorithms are trained on this data to predict LNP composition with improved performance, guiding the rational design of new candidates. This iterative and data‐driven workflow enables continuous refinement of predictive models and accelerates the discovery of clinically relevant LNP formulations. Created with BioRender.

Looking ahead, high‐throughput strategies and AI‐driven design have the potential to streamline the bench‐to‐bedside journey for LNP therapeutics. One direction is to develop truly predictive and even generative ML models that can propose new LNP formulations with a high likelihood of clinical success, rather than simply rationalizing known data. Many combinatorial lipid synthesis pathways remain laborious due to the lack of commercially available building blocks and tedious synthesis and purification steps, which hinder their systematic optimization and investigation of structure‐function relationships.^[^ ^183^ ^]^ Recent progress in generative modeling approaches now enables the design of novel ionizable lipid structures while providing synthetic feasibility. As an example, a deep generative model was trained to assemble ionizable lipids from commercially available molecular fragments and even facilitated the corresponding synthesis routes, ensuring that all proposed structures can be synthesized using standard laboratory chemistry.^[^ ^184^ ^]^ Similarly, generative adversarial network (GAN) models have been applied to create new lipid structures with tumor‐specific physicochemical properties (e.g., pKa 6.2–6.8) while applying retrosynthesis‐based filters to discard molecules that cannot be readily made.^[^ ^185^ ^]^ Remarkably, more than 90% of GAN‐generated lipids passed this filter, with predicted synthesis requiring no more than three reaction steps. Furthermore, advances in ML and AI, including deep neural networks and generative models, can also be trained to propose novel lipid formulations optimized for specific performance criteria (e.g., maximizing cell‐type‐specific LNP delivery while minimizing immunotoxicity). The next step is to create a closed‐loop formulation optimization cycle by integrating automated high‐throughput synthesis, characterization, and screening with AL/ML optimization algorithms (**Figure** **13**). In this self‐driving system, models propose a set of formulations predicted to improve therapeutic delivery to a target tissue, which are then rapidly synthesized and tested using high‐throughput workflows and techniques. The new results are then fed back to refine the model until optimal performance is achieved. Such self‐driving labs using this closed‐loop approach of LNP development have already been proposed. For instance, the AI‐Guided Ionizable Lipid Engineering (AGILE) platform integrates automated high‐throughput lipid synthesis and screening with deep neural networks to rapidly optimize LNP formulations.^[^ ^61^ ^]^ Using a one‐pot combinatorial chemistry approach and robotic liquid handlers, AGILE synthesized a large library of ≈1200 ionizable lipids in a single day, which were then formulated into LNPs and measured their mRNA delivery efficiency, generating a rich dataset to train the model. This self‐driving lab identified a superior lipid H9, which outperforms industry standards in mRNA delivery to HeLa cells, and discovered a lipid R6 optimized for macrophage delivery.^[^ ^61^ ^]^ Notably, AGILE's deep learning model, pre‐trained on a vast virtual library and fine‐tuned with experimental data, uncovers subtle structure–function, particularly, the influence of variations in lipid tail structure and carbon chain length on transfection efficiency. This example demonstrates how robotics and AI can synergistically accelerate LNP design, reducing development cycles from months to days. The LUMI‐lab platform developed by Cui et al., is another example of a closed‐loop self‐driving lab.^[^ ^186^ ^]^ In this system, a pre‐trained formulation model (a large‐scale molecular deep learning model) serves as the “brain” to propose new ionizable lipid structures, automated robotics swiftly manufacture and evaluate the candidate LNPs, and the resulting data continuously refine the model. Through ≈10 iteration cycles, LUMI‐lab evaluated 1781 distinct lipid candidates, achieving improved mRNA delivery in a target cell line. By the final iteration, over 50% of new lipids showed >10 fold higher transfection efficiency than initial candidates. This modular autonomous platform also exemplifies a self‐driving laboratory for nanomedicine development, integrating robotic synthesis, HTS, and AI‐driven optimization to accelerate discovery off optimal LNP formulations.

Finally, standardizing HTS and HTC protocols for LNP development is increasingly recognized as a critical need, particularly as the field is shifting toward data‐driven LNP design strategies. Standardization methods would improve cross‐study data comparability and support the integration of datasets for AI‐guided formulation and screening, helping to move beyond traditional trial‐and‐error approaches. However, current high‐throughput studies often report formulation and biological performance data in inconsistent formats, which limits their reusability and interpretability. For instance, lipid compositions may be reported as mole% or weight%, and physicochemical data such as scattering profiles are often normalized differently across studies. Metadata related to sample preparation techniques (e.g., storage, filtration methods, and buffer systems) are underreported or omitted entirely. Furthermore, variability in experimental conditions (e.g., cell lines, doses, and time points) further complicates cross‐platform comparisons. These inconsistencies significantly prevent data interoperability and limit the ability to use datasets for predictive modeling. Addressing these challenges requires the development of standardized metadata that describes well‐defined parameters and shared data repositories to ensure data interoperability and reproducibility. Initiatives are beginning to fill this gap. Promising examples include Kim et al.’s “Simple Scattering”, a platform for storing standardized small‐angle scattering data and associated metadata across different LNP formulations,^[^ ^157^ ^]^ and Sanofi's LipoBART, a GitHub repository that defines key structural and compositional features of high‐performing LNPs.^[^ ^187^ ^]^ These resources, when integrated with advanced predictive models such as TransMA,^[^ ^188^ ^]^ hold promise for building a robust, shared data ecosystem. This would not only streamline cross‐study comparisons but also support the training of more reliable AL/ML models for predictive lipid design, ultimately reducing reliance on empirical, trial‐and‐error workflows and accelerating rational LNP discovery.

## Conflict of Interest

The authors declare no conflict of interest.
